# *miR-9a* mediates the role of Lethal giant larvae as an epithelial growth inhibitor in *Drosophila*

**DOI:** 10.1242/bio.027391

**Published:** 2017-12-20

**Authors:** Scott G. Daniel, Atlantis D. Russ, Kathryn M. Guthridge, Ammad I. Raina, Patricia S. Estes, Linda M. Parsons, Helena E. Richardson, Joyce A. Schroeder, Daniela C. Zarnescu

**Affiliations:** 1 Department of Molecular and Cellular Biology, University of Arizona, Tucson, AZ 85721, USA; 2 Genetics Graduate Interdisciplinary Program, University of Arizona, Tucson, AZ 85721, USA; 3 Arizona Cancer Center, University of Arizona, Tucson, AZ 85721, USA; 4 Cell Cycle and Development Laboratory, Research Division, Peter MacCallum Cancer Center, Melbourne, Victoria 3000, Australia; 5 Department of Genetics, University of Melbourne, Melbourne, Victoria 3010, Australia; 6 Sir Peter MacCallum Department of Oncology, Department of Anatomy & Neuroscience, Department of Biochemistry & Molecular Biology, University of Melbourne, Melbourne, Victoria 3000, Australia; 7 Department of Biochemistry & Genetics, La Trobe Institute of Molecular Science, La Trobe University, Melbourne, Victoria 3086, Australia

**Keywords:** miRNA, Epithelial growth, *Drosophila*

## Abstract

*Drosophila lethal giant larvae* (*lgl*) encodes a conserved tumor suppressor with established roles in cell polarity, asymmetric division, and proliferation control. Lgl's human orthologs, HUGL1 and HUGL2, are altered in human cancers, however, its mechanistic role as a tumor suppressor remains poorly understood. Based on a previously established connection between Lgl and Fragile X protein (FMRP), a miRNA-associated translational regulator, we hypothesized that Lgl may exert its role as a tumor suppressor by interacting with the miRNA pathway. Consistent with this model, we found that *lgl* is a dominant modifier of Argonaute1 overexpression in the eye neuroepithelium. Using microarray profiling we identified a core set of ten miRNAs that are altered throughout tumorigenesis in *Drosophila lgl* mutants. Among these are several miRNAs previously linked to human cancers including *miR-9a*, which we found to be downregulated in *lgl* neuroepithelial tissues. To determine whether *miR-9a* can act as an effector of Lgl *in vivo*, we overexpressed it in the context of *lgl* knock-down by RNAi and found it able to reduce the overgrowth phenotype caused by Lgl loss in epithelia. Furthermore, cross-comparisons between miRNA and mRNA profiling in *lgl* mutant tissues and human breast cancer cells identified *thrombospondin* (*tsp*) as a common factor altered in both fly and human breast cancer tumorigenesis models. Our work provides the first evidence of a functional connection between Lgl and the miRNA pathway, demonstrates that *miR-9a* mediates Lgl's role in restricting epithelial proliferation, and provides novel insights into pathways controlled by Lgl during tumor progression.

## Abstract (precis)

**Summary:** Mir-9a overexpression can suppress the overgrowth phenotype caused by Lgl knock-down in epithelia. Gene profiling identifies pathways dysregulated in *lgl* mutants and shared features between flies and human cancer cells.

## INTRODUCTION

*lethal giant larvae* (*lgl*) encodes a conserved tumor suppressor with established roles in cell polarity and proliferation control (Cao et al., 2015; Elsum et al., 2012; Froldi et al., 2008; Grifoni et al., 2013; Humbert et al., 2008; Walker et al., 2006). Loss of *lgl* leads to invasive neural and epithelial tumors accompanied by lethality at the third instar larval stage in *Drosophila* (Beaucher et al., 2007; Calleja et al., 2016; Gateff, 1978; Merz et al., 1990; Woodhouse et al., 1998). Neural stem cells lacking functional *lgl* self-renew but fail to differentiate, resulting in stem cell tumors (Ohshiro et al., 2000; Peng et al., 2000). In various types of epithelial cells in *Drosophila*, *lgl*, along with *discs-large* (*dlg*) and *scribbled* (*scrib*), is involved in apico-basal polarity by controlling the appropriate localization of basolateral proteins and adherens junctions (Bilder et al., 2000). Although loss of polarity and overproliferation are separable, overall, in the absence of *lgl*, epithelial cells lose their monolayer structure as well as the ability to terminally differentiate and instead, overproliferate into neoplastic tumors with invasive characteristics (Froldi et al., 2010, 2008; Grzeschik et al., 2007; Humbert et al., 2008). In neural stem cells, Lgl has been shown to interact with and antagonize the atypical protein kinase C (aPKC)/PAR polarity complex to control apico-basal polarity and cell proliferation (Betschinger et al., 2003). Likewise, in epithelial tissues, Lgl and aPKC also have antagonistic functions in cell polarity and tissue growth (Bilder et al., 2003; Eder et al., 2005; Tanentzapf and Tepass, 2003). Recently, clonal analyses in the developing eye epithelia have demonstrated that Lgl loss downregulates the Salvador/Warts/Hippo tissue growth control pathway, as well as upregulates the Notch pathway, leading to ectopic cell proliferation and reduced apoptosis (Grzeschik et al., 2010b; Parsons et al., 2014; Portela et al., 2015). Moreover, in *lgl* mutant wing epithelial tissue, the dMyc transcription factor, and the Hippo, EGFR-Ras-ERK, PI3K-AKT, JNK, Jak-STAT and hypoxia signalling pathways are dysregulated (Grifoni et al., 2015). Additionally, another study showed that Hippo pathway targets, and components of the EGFR, Wingless and Decapentaplegic pathway are elevated, and differentiation is compromised in *lgl* mutant wing epithelial tissue (Khan et al., 2013).

*lgl* orthologs have been found in many different species including yeast, worms, zebrafish, mice, and humans (Strand et al., 1995). In mice and humans there are two paralogs each, known as *mlgl1/mlgl2* and *HUGL1*/*HUGL2*, respectively. The exogenous expression of the human protein, HUGL1, in flies can rescue the lethality caused by an *lgl* null mutation, which demonstrates functional conservation across species (Grifoni et al., 2004). Knock-out of the mouse ortholog, *mlgl1*, results in neuroectodermal tumors and neonatal lethality (Klezovitch et al., 2004), whereas knock-out of *mlgl2* causes a branching morphogenesis defect during placental development (Sripathy et al., 2011).

In recent years, aberrant localization and/or reduced expression for either HUGLl or HUGL2 have been reported in several epithelial cancers including cancer of the breast, stomach, colon, ovary, prostate, skin, endometrium, oesophageal, lung and glioma (Grifoni et al., 2004; Imamura et al., 2013; Kuphal et al., 2006; Lisovsky et al., 2009, 2010; Liu et al., 2015; Matsuzaki et al., 2015; Nam et al., 2014; Schimanski et al., 2005; Song et al., 2013; Spaderna et al., 2008; Tsuruga et al., 2007). In addition, the locus that contains *HUGL1* (at 17p11.2) is deleted in cases of medulloblastoma (Batra et al., 1995), in early stages of breast cancer (Johnson et al., 2012), and in chromosomally unstable colon cancers (Lassmann et al., 2007). These correlations suggest that in humans, Lgl orthologs may also act as tumor suppressors. Indeed, recent experiments with human breast cancer cells further support this notion, reporting transcriptional regulation of HUGL2 by SNAIL1 and revealing that HUGL2 is a driver of the mesenchymal to epithelial transition (EMT) (Kashyap et al., 2012). Recently, we demonstrated a role for both HUGL1 and HUGL2 in maintaining cell polarity and growth control in human mammary epithelium (Russ et al., 2012). We found that while HUGL1 and HUGL2 inhibited EMT, they also promoted anoikis and polarity in 3-dimensional cultures, as well as inhibited growth of breast cancer cells.

Using genetic interaction experiments in *Drosophila* we have previously identified *lgl* as a dominant modifier of Fragile X protein (FMRP) (Zarnescu et al., 2005), an RNA binding protein implicated in the microRNA (miRNA) pathway (Caudy et al., 2002; Ishizuka et al., 2002). FMRP exhibits physical and genetic interactions with Argonaute 1(AGO1), a core component of the miRNA machinery, which regulates the processing of mature miRNAs (Jin et al., 2004). Given the functional connection between Lgl and FMRP, we hypothesized that Lgl's tumor suppressor function, in addition to its effect on signaling pathways, might also involve regulation of miRNA expression. miRNAs are noncoding RNAs that can control gene expression by inhibiting mRNA translation or by degrading transcripts (Carthew and Sontheimer, 2009). Recently, a large body of evidence has emerged linking dysregulation of miRNA expression to the development and progression of tumors, with miRNAs acting as either oncogenes or tumor suppressors (reviewed in Ventura and Jacks, 2009). For example, *let-7* has multiple cancer-relevant mRNA targets, including those involved in proliferation, differentiation, and stem cell maintenance (reviewed in Boyerinas et al., 2010). This miRNA is highly conserved across species (Pasquinelli et al., 2000) and loss of its expression has been documented in many types of cancer, including breast cancer (Dahiya et al., 2008; O'Hara et al., 2009; Sempere et al., 2007; Takamizawa et al., 2004). *let-7* and several other miRNAs are currently being investigated for potential use as cancer therapeutics (Barh et al., 2010; Hwang and Mendell, 2006; Liu et al., 2008; Tavazoie et al., 2008; Volinia et al., 2006).

Here, using *Drosophila* as a model, we found that *lgl* loss-of-function mutations suppress the *AGO1* overexpression phenotype in the eye, consistent with a functional link between Lgl and the miRNA pathway. Next, we used microarray profiling to identify miRNAs that are misexpressed in neural and epithelial tissues including brain and eye-antennal imaginal discs at different stages of tumor growth in *lgl* loss-of-function mutants. *lgl* mutant larvae are normal sized at the onset of the third instar stage, however at the end of this developmental stage and while wild-type larvae enter pupation, *lgl* larvae continue to grow and accumulate large, invasive imaginal disc and brain tumors (Beaucher et al., 2007; Gateff, 1978; Woodhouse et al., 1998). Thus, the fly provides a unique model of *in vivo* tumorigenesis, whereby neural and epithelial tissues undergo transformation within a few days and importantly, recapitulate several features of metastasis (Beaucher et al., 2007; Calleja et al., 2016; Froldi et al., 2010; Grifoni et al., 2015; Woodhouse et al., 1998, 1994). We performed our profiling experiments at three different time-points: at tumor onset, during tumor growth, and later, during malignant progression. From these expression profiles, we identified several miRNAs that are dysregulated in *lgl* tumors. Notably, several of the miRNAs we found to be misexpressed in *lgl* mutant tissues have also been linked to human cancers, including *let-7* (Boyerinas et al., 2010), *miR-9a* (Hildebrandt et al., 2010; Lehmann et al., 2008; Zhang et al., 2012) and *miR-210* (Tsuchiya et al., 2011). To evaluate the physiological significance of our findings we began by testing whether *miR-9a* can modulate *lgl*'s phenotypes *in vivo*. Consistent with it being downregulated in *lgl* mutant tumors and functionally important for the *lgl* mutant phenotype, we found that *miR-9a* overexpression reduces the overgrowth phenotype caused by *lgl* loss of function in the wing epithelium. Although the precise mechanism of these genetic interactions remains to be established, here we provide the first evidence of a functional connection between Lgl and the miRNA pathway *in vivo*. Our data show that *miR-9a* mediates at least some aspects of Lgl's role in tumor suppression.

When comparing the miRNAs that are dysregulated throughout the tumorigenesis process, we identified a subset of ten miRNAs that are consistently misexpressed. This core set of miRNAs was further compared to mRNA expression changes in *lgl* mutant neuroepithelial tissues, late in tumorigenesis. Using cross-comparisons between miRNA and mRNA profiling data, we further identified a set of 38 mRNAs that are predicted to be *in vivo* targets of the core set of ten miRNAs dysregulated in *lgl* tumors. GO term and Cytoscape analyses of these mRNAs pinpoint to both established and novel pathways being involved in Lgl-mediated tumor progression. To further determine the significance of our findings in the fly model we identified mRNAs that are altered in an *in vitro* model of cancer based on HUGL1 knock-down in human breast epithelia. When compared with the gene expression profiling in the fly model, we found that *thrombospondin* (*tsp*) is a common factor altered between the fly and human models of tumorigenesis used in our studies. This finding underscores the significance of our combined approach and provides new insights into Lgl's role as a tumor suppressor.

## RESULTS

### *lgl* interacts genetically with the miRNA pathway

**Fig. 1.**
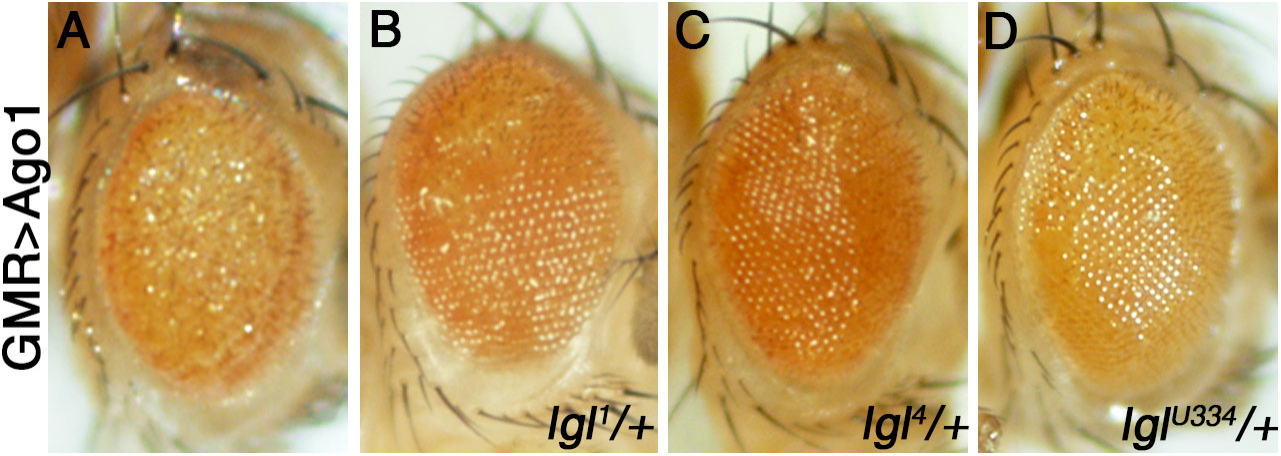
***lgl* interacts genetically with Argonaute 1 (AGO1) in the eye.** (A) Overexpression of *AGO1* using *GMR-Gal4* results in a rough eye phenotype. (B-D) Three independent alleles of *lgl*, namely *lgl^1^*, *lgl^4^* and *lgl^U334^*, can dominantly suppress the *GMR>AGO1* phenotype. Genotypes as indicated. *N*=at least 10 adults were imaged per genotype.

We have previously shown that Lgl and Fragile X protein (FMRP), an RNA binding protein known as a regulator of the miRNA pathway, form a functional protein complex (Jin et al., 2004; Zarnescu et al., 2005). These findings led us to hypothesize that Lgl may also be involved in regulating the miRNA pathway. To test this possibility, we conducted genetic interaction experiments between *ago1*, a core component of the miRNA machinery, and *lgl* in the *Drosophila* neural epithelium. Overexpression of *AGO1* in the developing retina was previously shown to generate a rough eye phenotype accompanied by depigmentation (Fig. 1A) (Jin et al., 2004). Here, using three independent alleles, i.e. *lgl^1^*, *lgl^4^* and *lgl^U334^*, we found that *lgl* loss-of-function mutations can dominantly suppress the eye phenotype caused by *AGO1* overexpression (Fig. 1). These data support our hypothesis and suggest that *lgl* may modulate the output of the miRNA pathway *in vivo*.

### Loss of *lgl* leads to misexpression of specific miRNAs in neuroepithelial tissues

**Fig. 2.**
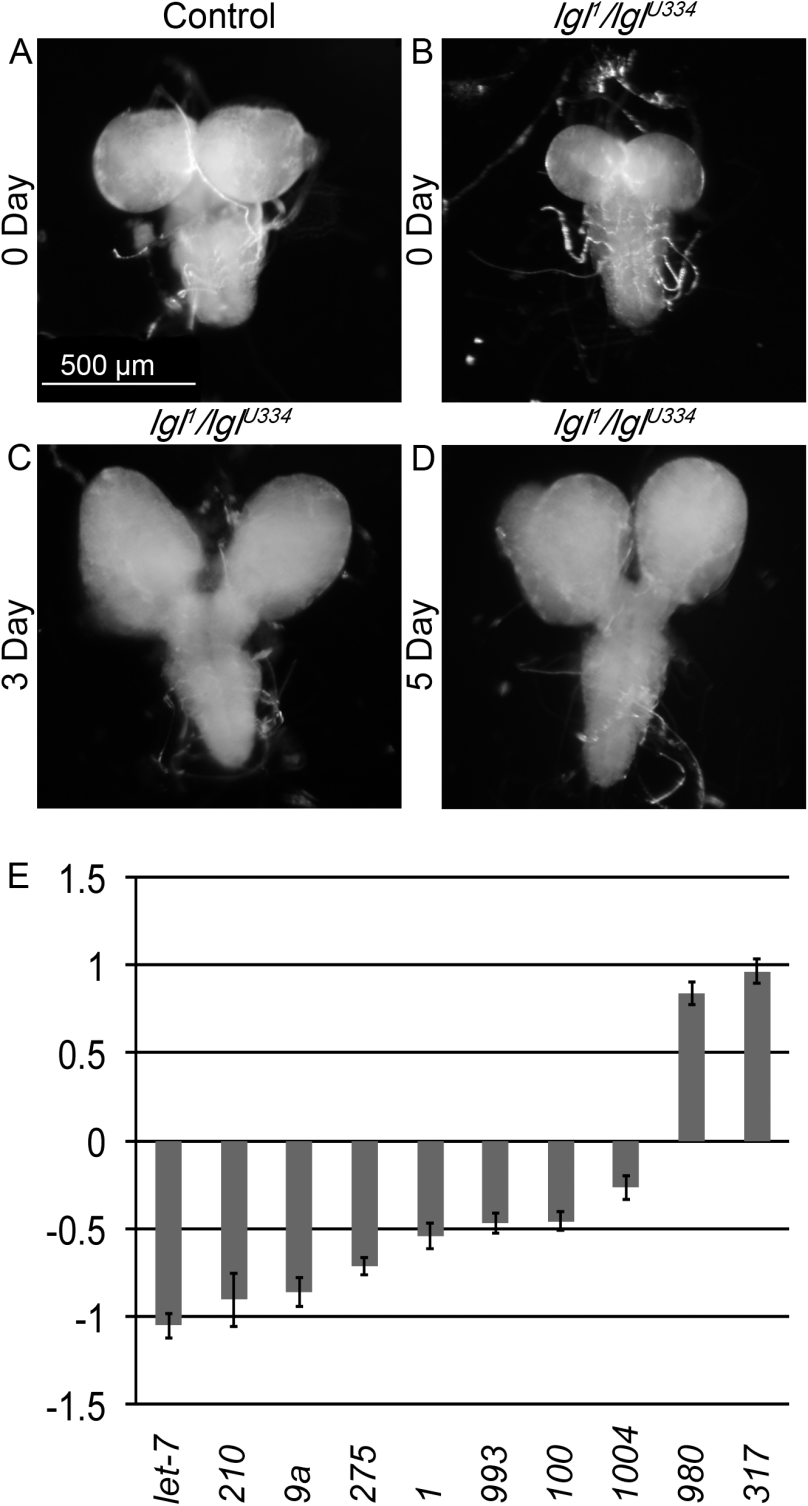
***lgl* brain tissue and dysregulated microRNAs.** (A-D) Cephalic complexes and ventral ganglia of third instar larvae. (A) Day 0 of control's third instar. (B-D) *lgl* mutants do not pupate as normal, day 0, 3, and 5 of *lgl* third instar are shown. (E) Graph of up- and down-regulated microRNAs from microarrays done on tissue from the brains shown in (A-D). Log of fold-change (logFC) is estimated from a linear model of the expression values as computed by the microarray analysis package, limma. The microRNAs listed here, represent ones that were dysregulated across all time-points for mutants, when compared to controls. All microRNAs shown were found to be significantly dysregulated with *P*<0.05, Benjamini-Hochberg multiple testing correction. Error bars show standard error of the mean.

Given the genetic interactions between Lgl and components of the miRNA pathway (Fig. 1), we sought to identify miRNAs that are misexpressed in lgl mutant tissues and thus may provide novel insights into Lgl's function as a tumor suppressor. To this end, we dissected larval cephalic complexes (i.e. brains and the eye-antennal imaginal discs, which undergo transformation due to loss of lgl) from third instar larvae at three different time points of relevance to the tumor progression process *in vivo*. The first time-point corresponds to the late third instar larval stage (Day 0 in our study), when lgl mutant tissues appear relatively normal, with no signs of overproliferation or loss of polarity compared to wild-type (Fig. 2A,B). For the second time-point, we analyzed lgl mutant larvae three days later (Day 3 in our study) when they appear overgrown and their tissues exhibit visible malformations (Fig. 2C). The third and final time point was chosen after five days (Day 5 in our study) when lgl mutants appear grossly bloated and are filled with tumors that eventually kill the larvae (see Fig. 2D for cephalic complexes at Day 5). Since normal larvae enter pupation after 24 h in the third instar stage, for the second and third time points no wild-type controls were available for comparison, thus the Day 0 wild-type was used as a control throughout. In these studies we compared lgl^1^/lgl^U334^ mutants to a genomic rescue line as wild-type control (P[lgl^+^];lgl^1^/lgl^U334^).

To identify miRNAs that are misexpressed in lgl mutant tissues both before and after the onset of aberrant tissue growth, we probed miRNA microarrays with labeled miRNAs isolated from lgl mutants or controls (see Materials and Methods). Three biological replicates (three technical replicates each) were performed. After normalization, differences in the expression of miRNAs were fitted to a linear model that was then used to calculate fold change and statistical significance. Significantly dysregulated miRNAs were determined based on a *P* value cut-off of 0.05, calculated using the Benjamini-Hochberg multiple testing correction (Reiner et al., 2003). Out of 147 miRNAs, we found 38 miRNAs dysregulated in lgl mutants compared to controls at Day 0, 22 at Day 3 and 58 at Day 5 (Table S1). Of these, a core set of 10 miRNAs was found to be consistently dysregulated across all time points: let-7, miR-210, miR-9a, miR-275, miR-1, miR-993, miR-100, miR-1004, miR-980 and miR-317 (Fig. 2E). For validation, the expression levels of let-7, miR-210 and miR-9a in Day 0 lgl mutant tissues were compared to controls using Real-Time PCR with small RNA U6 as a housekeeping gene (data not shown). Expression of all three miRNAs corroborated with the microarray results showing significantly lowered expression in mutant tissues (Table S1). Overall, the majority of altered miRNAs corresponded to mature rather than precursor forms, suggesting that Lgl is not involved in regulation of these targets at a transcriptional level.

Although at this point we do not know whether Lgl regulates the processing of these miRNAs directly or indirectly, via RNA binding proteins such as FMRP or AGO1, our data indicate that loss of *lgl* leads to the dysregulation of specific miRNAs in a temporal manner that corresponds to critical stages of tumor progression including initiation, growth and malignant progression. Furthermore, our findings define an Lgl*-*specific ‘signature’ represented by a core set of ten miRNAs that are dysregulated throughout tumorigenesis and may help elucidate the mechanisms by which Lgl acts as a tumor suppressor.

### miR-9a overexpression rescues the overgrowth phenotype of lgl knock-down in the wing epithelium

**Fig. 3.**
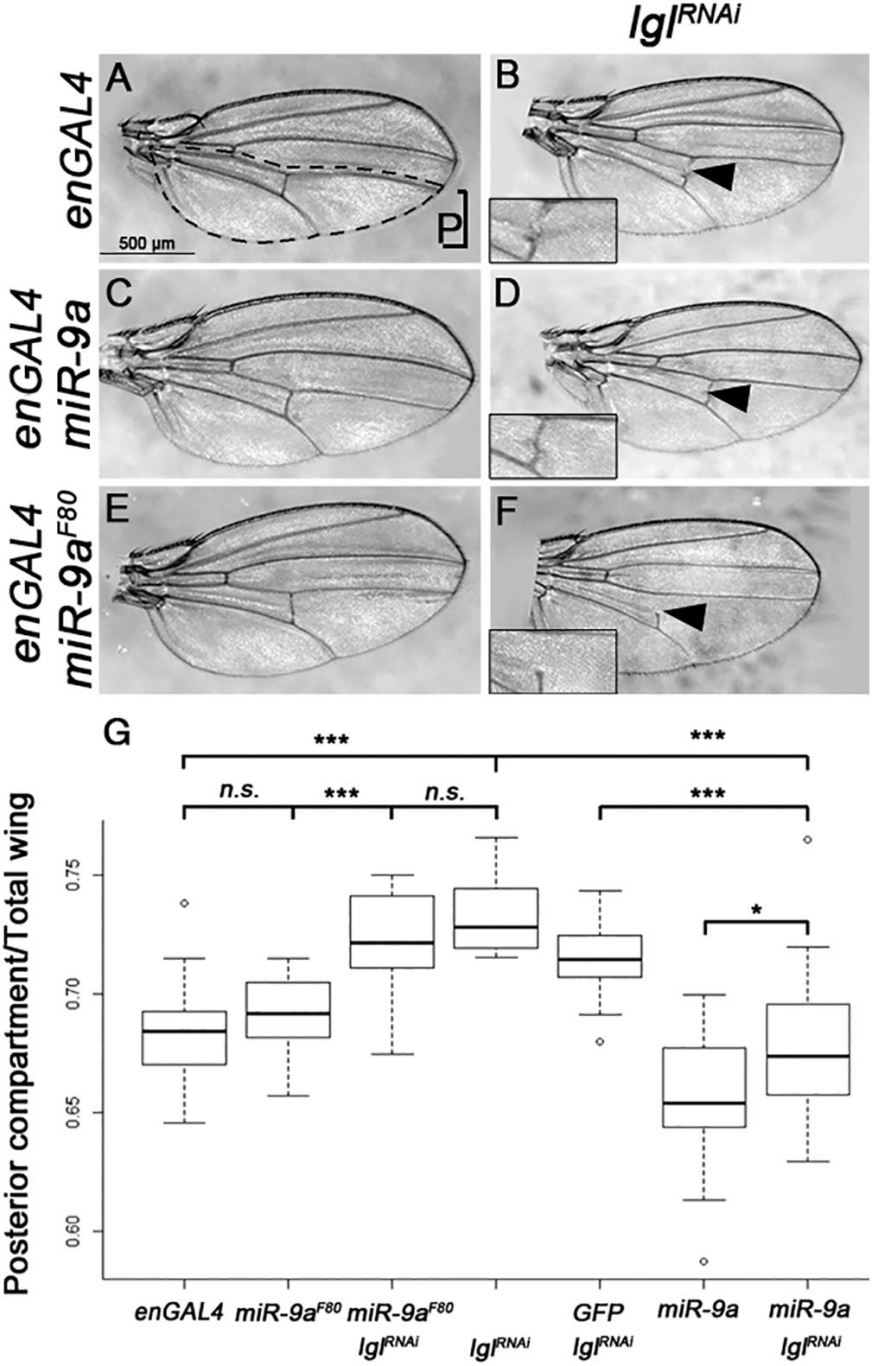
**miR-9a and lgl exhibit genetic interactions in the wing epithelium.** (A) *en-GAL4* control, posterior compartment P as indicated; (B) *en-GAL4; UAS-lgl^RNAi^*; (C) *en-GAL4; UAS-miR-9a*; (D) *en-GAL4; UAS-lgl^RNAi^/UAS-miR-9a*; (E) *en-GAL4; miR-9a^F80^/+*; (F) *en-GAL4; UAS-lgl^RNAi^/miR-9a^F80^*. Insets show incomplete cross-vein phenotype. (G) Graph of posterior wing region area divided by total wing area. Genotypes as indicated. ***P<0.001, *P<0.05, n.s., not significant; student's *t*-test was used to determine statistical significance. For number of wings analysed, see Materials and Methods. Box and whisker plots show median, upper and lower quartiles, highest and lowest points, and outliers.

One of the premises of our work is that the miRNAs found to be dysregulated in *lgl* mutant tissues may mediate Lgl's function as a tumor suppressor. Thus, we hypothesize that restoring the expression of these miRNAs in an *lgl* mutant background might reduce the severity of the mutant phenotype. We began to test this hypothesis by asking whether *miR-9a* or *let-7* overexpression can mitigate *lgl* loss-of-function phenotypes. For these experiments we focused on the wing, where Lgl knock-down by RNAi using the engrailed driver (*en-GAL4*) causes epithelial overgrowth accompanied by an increase in the posterior compartment as compared to the total wing area (with the posterior region defined as wing area posterior to the longitudinal vein L4, see dashed outline in Fig. 3A (Parsons et al., 2017). Additional phenotypes caused by *lgl-RNAi* when driven in the posterior compartment by *en-GAL4* include incomplete cross veins (see insets in Fig. 3B,D and F) and tissue loss, usually near longitudinal vein L4 (data not shown). We could not pursue the *lgl–let-7* interaction due to lethality caused by *let-7* overexpression using *en-GAL4*. Therefore, we focused our studies on the *lgl–miR-9a* functional relationship. Using the *en-GAL4* driver, we overexpressed *miR-9a* in the context of *lgl* knock-down by RNAi and found a statistically significant reduction of the posterior compartment overgrowth caused by *lgl* loss (0.68±0.01 in *en-GAL4; UAS-lgl^RNAi^/UAS-miR-9a* compared to 0.732±0.004 in *en-GAL4; UAS-lgl^RNAi^*, *P*_value_=2.04×10^−7^; see Fig. 3B,D and G). Although overexpression of *miR-9a* alone caused a slight reduction in the wing posterior compartment area compared to *en-GAL4* controls (0.66±0.03 in *en-GAL4; UAS-miR-9a* compared to 0.68±0.02 in *en-GAL4*; see Fig. 3A,C and G), these findings demonstrate that *miR-9a* overexpression is sufficient to significantly reduce the epithelial overgrowth phenotype caused by *lgl* knock-down in the posterior compartment of the wing. To address potential concerns that the suppression by *miR-9a* may be due to a decrease in GAL4 activity caused by additional *UAS* elements controlling both *miR-9a* and *lgl^RNAi^* transgenes, we also compared *en-GAL4 UAS-GFP*; *UAS-lgl^RNAi^* to *en-GAL4; UAS-lgl^RNAi^*/*UAS-miR-9a* and found a similar suppressing effect (0.715±0.003 versus 0.68±0.01, *P*_value_=2.64×10^−5^; data not shown). These findings indicate that the overgrowth suppression we detected is not due to a reduction in GAL4 activity but rather due to *miR-9a* expression in the context of *lgl* knock-down. In contrast, *miR-9a* reduction using a loss-of-function allele, *miR-9a^F80^*, had no significant effect on posterior compartment size, either on its own (0.68±0.02 in *en-GAL4* compared to 0.69±0.02 in *en-GAL4*; *miR-9a^F80^*/+) or in the context of *lgl^RNAi^* (0.722±0.004 in *en-GAL4; UAS-lgl^RNAi^/miR-9a^F80^* compared to 0.732±0.004 in *en-GAL4; UAS-lgl^RNAi^ P*_value_=0.09). The failure to observe a genetic interaction in the *miR-9a^F80^* heterozygous background might indicate that *miR-9a* is abundantly expressed, and reducing its dosage by ∼50% is not sufficient to significantly alter the tissue growth defects due to *lgl* knock-down. To determine whether *miR-9a* overexpression mitigates growth and/or apoptosis defects caused by *lgl* knock-down, we quantified the wing disc size and caspase intensity in third instar wing discs (Fig. S1). These experiments showed a significant increase in caspase activity in the wing pouch, within the en-GAL4 domain for *lgl^RNAi^* compared to controls (Fig. S1A-G). However, *miR-9a* overexpression did not have a suppressing effect, and the size of the posterior compartment was comparable for *lgl^RNAi^ miR-9a* and *lgl^RNAi^* wing discs (Fig. S1A-E,F) suggesting that *miR-9a* exerts its suppressing effect on *lgl^RNAi^* during morphogenesis. Nevertheless, these data provide the first *in vivo* evidence that restoring *miR-9a* expression in epithelia can rescue the overgrowth phenotype due to Lgl knock-down. Importantly, these findings are consistent with the miRNA profiling data and support our hypothesis that miRNAs may act as effectors of Lgl's tumor suppressor function in epithelia.

### *In silico* identification of mRNAs targets for the miRNAs dysregulated in lgl mutant tissues

A major challenge in the miRNA field is to identify *in vivo* target transcripts. Since individual miRNAs can target several mRNAs at once, it is imperative that these transcripts are identified, which should lead to a better mechanistic understanding of the miRNA pathway and the development of therapeutic strategies for diseases linked to miRNA dysregulation. Several software packages have been developed for predicting mRNA targets of miRNAs, however cell-based assays have shown that about half of the computational predictions do not validate *in vivo* (Chiang et al., 2010). To address this important issue, we complemented our miRNA microarray data with mRNA profiling experiments. To generate mRNA expression profiles, we compared *lgl* mutant cephalic tissues dissected from four day old (Day 4) 3rd instar *lgl* null mutant (*lgl^27S3^/lgl^E2S31^*) (Grzeschik et al., 2007) larvae to wild-type control tissues (*w^1118^* larvae at Day 0). After background correction and normalization, any differences in mRNA expression were fitted to a linear model that was then used to calculate fold change and significance of dysregulation. Using a *P* value cut-off of 0.05 and a logFC cut-off of 1, we identified 169 mRNAs that were significantly dysregulated in mutant tissues versus wild-type (Table S2). For the mRNA microarray data, *P* values were adjusted using Benjamini-Hochberg multiple testing correction (Reiner et al., 2003). Next, using the miRNA targeting algorithm miRanda (Enright et al., 2003) as implemented by microRNA.org (Betel et al., 2008), we matched the core set of 10 misexpressed miRNAs to the 169 dysregulated mRNA transcripts identified (John et al., 2004).

**Fig. 4.**
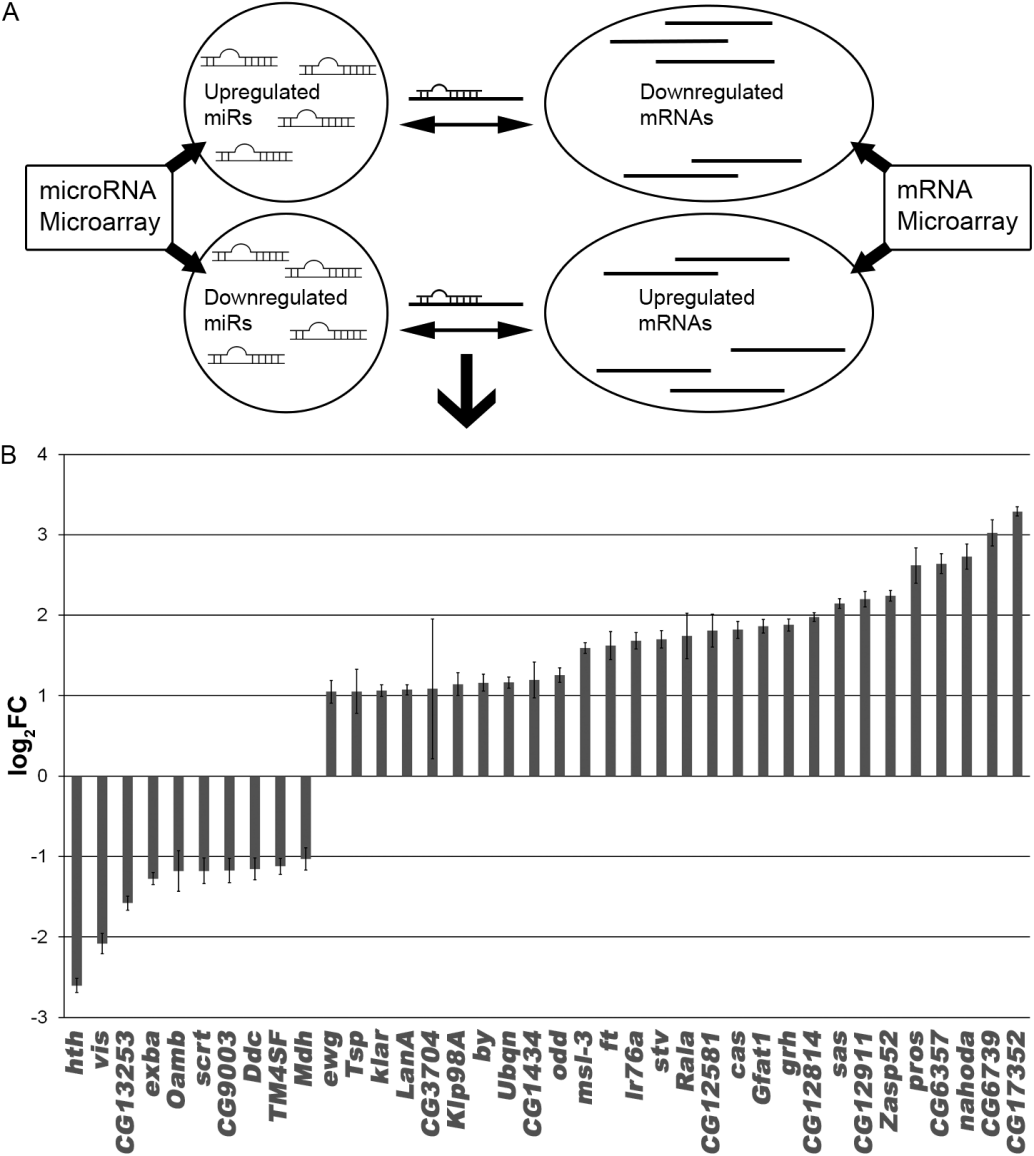
**microRNA targeting strategy and dysregulated mRNAs.** (A) Significantly dysregulated microRNAs were matched to mRNAs based on predictions given by the miRanda algorithm. Additionally, matches were validated by their direction of deregulation; upregulated miRNAs matched downregulated mRNAs while downregulated miRNAs matched upregulated mRNAs. (B) The matching mRNAs after applying our targeting strategy (see text). Log of fold-change (logFC) is estimated from a linear model of the expression values as computed by the microarray analysis package, limma. All mRNAs shown were found to be significantly dysregulated with *P*<0.05, Benjamini-Hochberg multiple testing correction. Error bars show standard error of the mean.

**Fig. 5.**
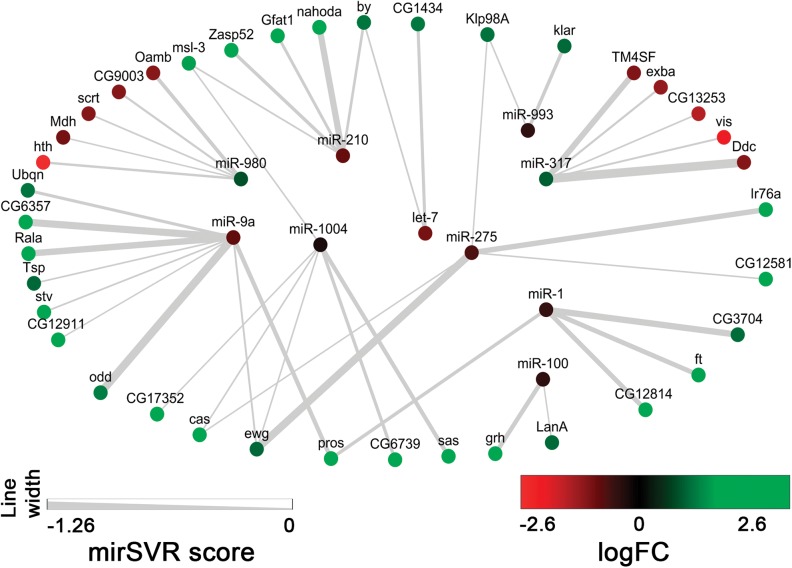
**microRNA targeting network.** MicroRNAs (center) target multiple mRNAs (outer ring); in turn, some mRNAs are targeted by a number of microRNAs (e.g. *Klp98A* is targeted by *miR-993* and *miR-275*). The spectrum of maximum downregulation (−2.6 logFC) to maximum upregulation (3.3 logFC) is denoted by a standard red to green gradient. Only upregulated mRNAs are targeted by downregulated microRNAs, while downregulated mRNAs are targeted by upregulated microRNAs. Width of lines connecting microRNA to mRNA represents strength of targeting, as defined by the mirSVR score of the miRanda targeting algorithm. mirSVR score heat map as shown. Green is upregulated, and red is downregulated miRs or genes.

Given the widely accepted paradigm of mRNA translational repression and mRNA stability by miRNAs (Carthew and Sontheimer, 2009), we matched those miRNAs that were upregulated to predicted mRNA targets that were downregulated (Fig. 4A). Conversely, miRNAs that were found to be downregulated in *lgl* tissues were matched to upregulated mRNAs. This matching approach allowed us to filter our data and discard: (1) the upregulated mRNAs predicted by microRNA.org to be targeted by upregulated miRNAs, and (2) the downregulated mRNAs predicted to be targeted by downregulated miRNAs. Of the 112 miRNA-mRNA matches predicted (including matches to different sequences within the 3′ UTR for the same gene), 50.9% (57 of 112) were judged to be parsimonious by the filtering method we implemented. Using this approach, we found 38 mRNAs that were both inversely correlated with our core set of ten miRNAs and predicted by microRNA.org to be direct targets (Fig. 4B). For example, *miR-980* and *miR-317*, which were found to be upregulated in *lgl* tissues, matched 10 downregulated mRNAs. The remaining eight miRNAs, which were downregulated, matched 28 upregulated mRNAs. The strength of the miRNA targeting (mirSVR score, as computed by microRNA.org) as well as the logFC of miRNAs and mRNAs was visualized using Cytoscape (Fig. 5). Notably, our bioinformatics analyses combined with miRNA and mRNA profiling indicate that among the genes identified there are several that have been previously linked to *lgl* function via standard molecular genetic approaches; *Prospero* (*pros*), *grainyhead* (*grh*) and *castor* (*cas*) are required for proper proliferation and differentiation of neural stem cells, which *lgl* has also been demonstrated to control (Almeida and Bray, 2005; Bello et al., 2006; Betschinger et al., 2003, 2006; Klezovitch et al., 2004). Interestingly, *ft*, a transcriptional target of Yki in the Hippo pathway, which is deregulated in *lgl* mutant tissue (Grifoni et al., 2015; Grzeschik et al., 2010b; Khan et al., 2013), was upregulated in *lgl* mutant tissue, and is a predicted target for *miR-1*, suggesting that post-transcriptional regulation of Hippo pathway genes might also be controlled by Lgl.

### miRNA and mRNA targets are significantly enriched for GO terms related to hallmarks of cancer

Next, we analyzed the 10 miRNAs and the 38 mRNAs they targeted for gene ontology (GO) terms linked to cancer-related processes using the Bingo plug-in for Cytoscape (Maere et al., 2005). Significantly enriched GO terms linked to cancer as determined by processes associated with the disease include cell polarity (e.g. basolateral plasma membrane), cell-cell junctions (e.g. cell-substrate adherens junction, cell-substrate junction), cellular proliferation and differentiation (e.g. ganglion mother cell fate determination, neuron fate commitment, cell fate commitment, etc.) (Hanahan and Weinberg, 2011) (see Table 1). Most notable are cell fate commitment and neuron differentiation, each with eight genes associated. Additional GO terms that were significantly associated with our set of genes include various aspects of development (Table S3). Interestingly, this matching analysis further confirmed cellular processes that have been previously linked to *lgl* loss, such as ganglion mother cell fate determination, cell fate commitment, and basolateral polarity control (Bilder et al., 2000; Khan et al., 2013; Musch et al., 2002; Ohshiro et al., 2000; Peng et al., 2000; Russ et al., 2012).

**Table 1.**
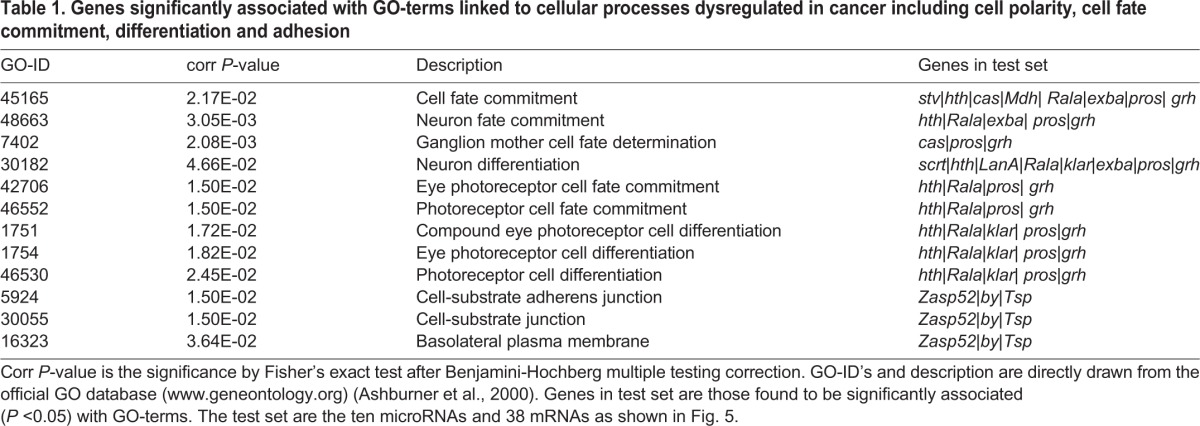
**Genes significantly associated with GO-terms linked to cellular processes dysregulated in cancer including cell polarity, cell fate commitment, differentiation and adhesion**

To predict human cancer pathways potentially affected by absence of HUGL1, we searched Flybase, Genecards miRBase and Ensembl online databases (www.flybase.org, www.genecards.org, www.ensembl.org) for human orthologs of *Drosophila* miRNAs and targets predicted to be altered due to loss of *lgl* (Crosby et al., 2007; Flicek et al., 2008; Safran et al., 2010). Interestingly, of the 14 genes we analyzed, five have human orthologs with a documented involvement in processes directly or closely linked to cancer (see Table 2; note several similar changes in mRNA expression between brains and wings). Additionally, five of the core set of ten miRNAs matched orthologous human sequences involved in carcinogenesis (see Table 3).

**Table 2.**
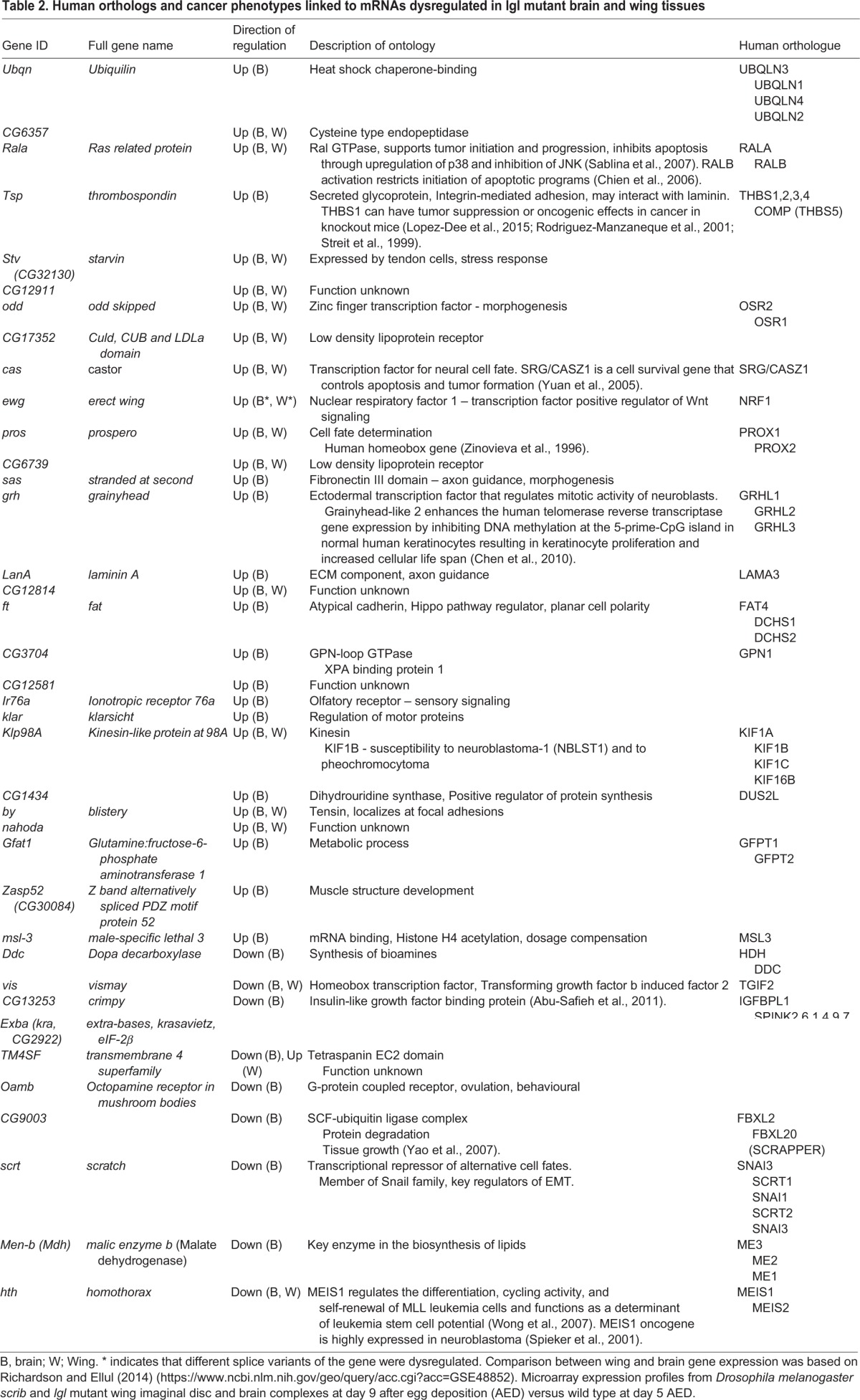
**Human orthologs and cancer phenotypes linked to mRNAs dysregulated in lgl mutant brain and wing tissues**

**Table 3.**
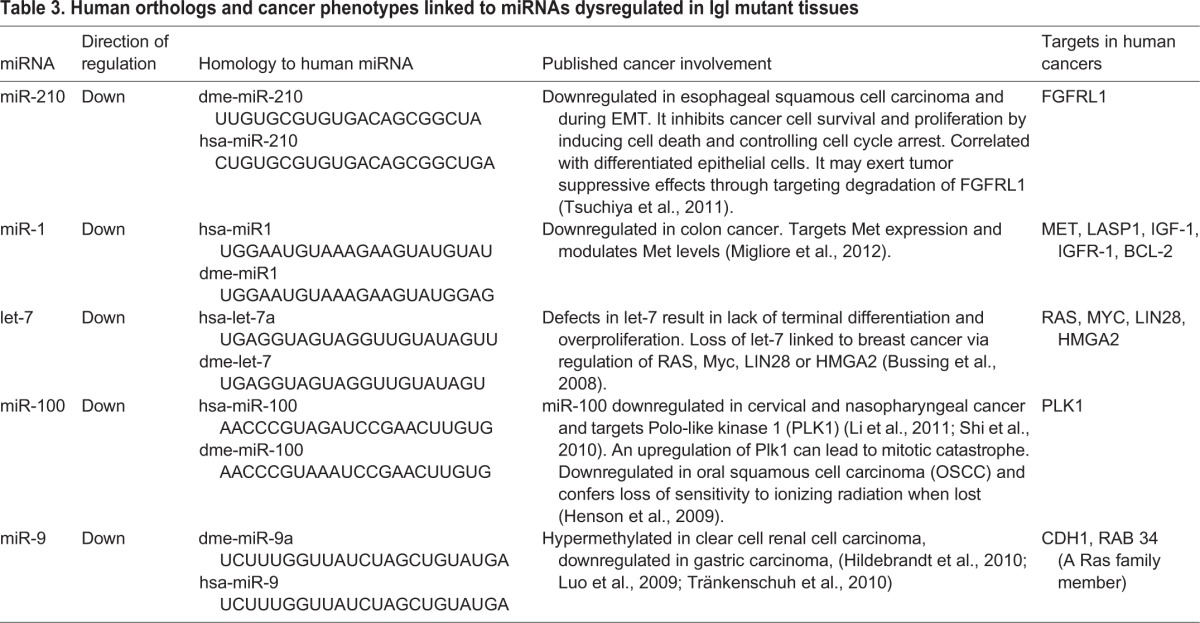
**Human orthologs and cancer phenotypes linked to miRNAs dysregulated in lgl mutant tissues**

### Loss of HUGL1 in human epithelial cells results in upregulation of transcripts linked to breast cancer

To probe the significance of our mRNA and miRNA profiling results for human cancers, we next performed knock-down of HUGL1 in the human mammary epithelial cell line, MCF10A. To silence HUGL1 expression, shRNA sequences designed against *HUGL1* mRNA were optimized in MCF10A cells. Two shRNAs resulted in optimal HUGL1 knock-down and were used in our experiments (Fig. S2). As we have recently shown, loss of HUGL1 alone in MCF10 cells is sufficient to induce overproliferation and loss of apico-basal cell polarity (Russ et al., 2012). The mRNA expression profiles of HUGL1 knock-down cells and shRNA control cells were assessed using a Real Time PCR array (SA Biosciences) containing 84 genes involved in breast cancer. These experiments identified five mRNAs that were significantly upregulated in the HUGL1 knock-down cells as compared to the controls (*ABCG2*, *ESR1*, *KRT19*, *MMP2*, *THBS1*, see Table 4). Notably, one of these genes is *THBS1* (*thrombospondin*), which was also identified as an upregulated mRNA in the *Drosophila lgl* mutant tissues and is predicted to be a target of *miR-9a* (see *tsp1* in Fig. 5). These findings underscore the importance of our combined bioinformatics and genetic approach to identify critical genes involved in tumorigenesis driven by Lgl. Future experiments will focus on the significance of THBS1 in human tumors characterized by HUGL1 loss.

**Table 4.**
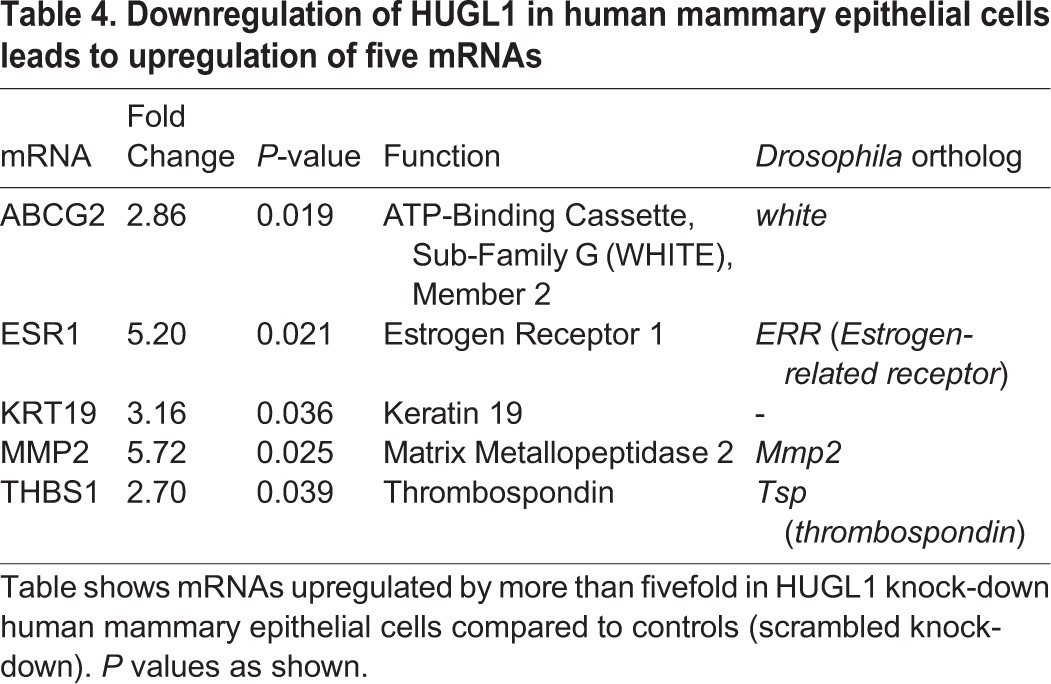
**Downregulation of HUGL1 in human mammary epithelial cells leads to upregulation of five mRNAs**

## DISCUSSION

Following our previous findings that Lgl regulates the RNA binding protein FMRP (Zarnescu et al., 2005), here we report a novel functional connection between the tumor suppressor Lgl and the miRNA pathway. Although miRNA dysregulation has been linked to cancer progression, the role of this pathway in tumorigenesis remains poorly understood (Iorio and Croce, 2012). As the human orthologs of Lgl, HUGL1 and/or HUGL2, are reported to be downregulated in breast and other epithelial cancers, we turned to the genetically tractable model *Drosophila* to explore the effects of *lgl* loss on the miRNA transcriptome and to identify miRNAs that may act as effectors of *lgl*'s ability to protect against cancer progression by modulating pathways involved in tumorigenesis including cell polarity, proliferation, differentiation, adhesion, cell fate and stem cell expansion.

First, by demonstrating a genetic interaction between *lgl* and *AGO1*, we identified a potential role for Lgl in the microRNA pathway. Next, to identify specific miRNAs misexpressed upon loss of *lgl*, we conducted miRNA microarrays to compare the expression levels of 147 miRNAs in *lgl* mutants compared to wild-type rescue larvae. Through this approach, we identified several miRNAs affected by *lgl* loss during tumor progression (38 for day 0, 32 for day 3, and 75 for day 5). Interestingly, only ten miRNAs were consistently affected across all time points studied. This time series enabled us to determine changes in miRNA expression before and during early- as well as late-tumor development that may serve in the future for biomarker development. This approach also provided a robust means for identifying a core set of ten miRNAs that define an Lgl tumor ‘signature’. Of the ten *Drosophila* miRNAs identified, *let-7*, *miR-210*, *miR-1*, *miR-100*, and *miR-9a* have homologues in humans as evidenced in miRBase (Griffiths-Jones et al., 2006). These same five have already been shown to exhibit tumor suppressive properties in human cancers (Henson et al., 2009; Iorio and Croce, 2012; Rothé et al., 2011; Volinia et al., 2006; Yu et al., 2007). Among these, *let-7* and *miR-100* are processed from the same primary miRNA.

*miR-9a*, a known growth regulator (Epstein et al., 2017; Suh et al., 2015), was found to be downregulated in the *lgl* mutant larval epithelial and neural tissues, therefore, we hypothesized that overexpressing *miR-9a* in *lgl* loss-of-function tissues may have a rescuing effect. By restoring levels of *miR-9a* in the wing, flies showed a statistically significant reduction in the overgrowth of the posterior compartment of the wing due to *lgl* knock-down.

We conducted two separate array experiments, one for miRNA and one for mRNA using two different *lgl* mutants. Furthermore, we compared our data to matches predicted by microRNA.org, which uses a machine-learning algorithm to score matches based on sequence similarity, free energy of the RNA duplex, and conservation of the target site. Target-matching algorithms have an estimated 50% error rate and indeed, using one of the latest target matching algorithms implemented by microRNA.org, miRanda, we discovered that the error rate was corroborated by our *in vivo* data. Thus, the power of our combined approach is that of target matches predicted by microRNA.org corresponding with an inverse expression relationship of miRNAs and their predicted mRNA targets in *lgl* mutant tissues (e.g. an upregulated miRNA validates a predicted mRNA target if that mRNA is downregulated). From this analysis, we could link dysregulated miRNAs with mRNAs in *lgl* mutant tissue; in particular, since we have shown that *miR-9a* deregulation contributes to the *lgl* mutant phenotype, these comparisons identify potential protein targets that are important in tumorigenesis upon Lgl depletion.

Cancer is a complex disease affecting many biological processes including: cell growth and proliferation, cell differentiation, angiogenesis, apoptosis, and genomic stability (Hanahan and Weinberg, 2011; Scheel and Weinberg, 2012). The dysregulated miRNAs and mRNAs in our analysis not only corroborated 3′ UTR targeting predicted by microRNA.org, but targeted mRNAs were significantly associated with GO-terms linked to cellular processes involved in cancer, including cell fate commitment, differentiation, and cell adhesion. Indeed, a disruption in differentiation of neuroblasts has been shown to result in brain tumors, a well-established phenotype of *lgl* mutants (Caussinus and Gonzalez, 2005; Gateff, 1978). Also cell adhesion GO-terms are highly relevant to tumorigenesis, since disruption of cell adhesion is associated with EMT and is critical for cells to break away from the epithelium and become invasive. Future studies will address the contribution of these genes, particularly the *miR-9a* targets, to the *lgl* mutant phenotype.

We have shown that knock-down of HUGL1 in human mammary epithelial cells leads to upregulation of five transcripts that have been linked to cancer stem cells, side population (SP) cells, or increased invasion in cancers (*ABCG2*, *MMP2*, *ESR1*, *THBS1* and *KRT19*; see Table 4). Of these, *ABCG2* (also known as breast cancer resistance protein) is an ATP-binding cassette transporter associated with the cancer stem cell phenotype and chemotherapeutic resistance, including therapy-refractory breast cancer (Ding et al., 2010; Doyle et al., 1998; Zhou et al., 2001). Although two miRNAs, *miR-328* and *miR-519c*, that have been previously described to downregulate human *ABCG2* (Pan et al., 2009; To et al., 2009) have no fly orthologs, we report a reduction in *miR-100*, which is also predicted to bind to the human *ABCG2* 3′UTR (To et al., 2008). Notably, the mature miRNA sequences of *miR-100* have a one base pair difference between human and *Drosophila* as reported by miRBase (Griffiths-Jones et al., 2006). Of the other four mRNAs significantly upregulated in mammary epithelial cells upon HUGL1 knock-down, *MMP2*, a matrix metalloprotease (MMP), is elevated in EMT (Laffin et al., 2008). Upregulation of MMPs has been previously observed in *Drosophila lgl* mutants, conferring invasive abilities, and has also been documented in cancer stem cells (Beaucher et al., 2007; Cronwright et al., 2005; Grifoni et al., 2015; Huang et al., 2011; Na et al., 2011; Woodhouse et al., 1994). In addition, *ESR1* is associated with aggressive breast tumor types, and *KRT 19* has been implicated as a marker of circulating tumor cells (Kim et al., 2011). Interestingly, *THBS1*, thrombospondin which regulates remodeling of the extracellular matrix (Huang et al., 2017), is an orthologue of *tsp*, a fly mRNA that we report upregulated in *lgl* mutants. This upregulation is potentially due to the reduction in *miR-9a* levels we detected, as *tsp* is a predicted target of this miRNA (Sampson et al., 2007; Selbach et al., 2008).

*let-7*, the most significantly downregulated miRNA in *lgl* tissues, has been shown to inhibit breast cancer cell proliferation in severely compromised immunodeficient (SCID) mice while its loss led to increased stem cell renewal (Yu et al., 2007). It was also shown to act as a repressor of stemness and is frequently lost in transformation (reviewed in Büssing et al., 2008). Similarly, in *Drosophila*, *let-7* is associated with cell differentiation and is regulated by the steroid hormone receptor, EcR (Caygill and Johnston, 2008; Kucherenko et al., 2012; Wu et al., 2012), and therefore its down-regulation would be expected to result in the accumulation of cells in a progenitor-like state. *miR-9* is downregulated in human gastric carcinoma, breast cancer, and ovarian cancer, and has been shown to exhibit control over cell proliferation and metastasis (Laios et al., 2008; Lujambio et al., 2008; Luo et al., 2009; Selcuklu et al., 2012). The findings presented here, coupled with an established role for Lgl as a regulator of stem and epithelial cell integrity in *Drosophila*, suggest that similar miRNAs and downstream pathways may be dysregulated in both fly and human tumors with loss of Lgl or HUGL1/2, respectively. We speculate that the contribution of *lgl* as a tumor suppressor may be attributed to its control over epithelial cell plasticity and localization of cell fate determinants, and/or by conferring protection against a dedifferentiated cancer stem cell population. Proper regulation of miRNAs *let-7* and *miR-9* by *lgl* via modulation of miRNA processing could contribute to this role.

In summary, we used a combined approach including bioinformatics in flies and human cells lacking Lgl and identified a ‘signature’ set of miRNAs characteristic to Lgl tumors. Cross comparisons between miRNA and mRNA profiling uncovered a small set of mRNAs that are both dysregulated *in vivo* and represent putative targets of the signature miRNAs. Although Lgl has been implicated in regulating endocytosis (Parsons et al., 2014; Portela et al., 2015) and non-muscle Myosin (Strand et al., 1994), our study suggests that Lgl might also regulate through its binding to FMRP (Zarnescu et al., 2005) the level of specific microRNAs, which would then affect the expression of various mRNAs including those involved in signaling pathways known to be deregulated by *lgl* impairment (Grifoni et al., 2015; Grzeschik et al., 2010a; Khan et al., 2013; Parsons et al., 2014). Among the dysregulated mRNAs, thrombospondin, a component of the extracellular matrix, was found to be misexpressed in both flies and human cells lacking Lgl. It is tempting to speculate that this connection between Lgl depletion and thrombospondin upregulation points to a mechanism involving the remodeling of the extracellular matrix, a key player in metastasis, which will be explored in future experiments. These results, together with genetic interaction experiments in *Drosophila*, suggest the potential for using miRNAs as therapeutics in tumors with Lgl loss.

## MATERIALS AND METHODS

### *Drosophila* genetics

All flies were raised on standard fly food at 25°C, except where otherwise noted. *lgl* alleles were previously described (Grzeschik et al., 2007; Zarnescu et al., 2005). *lgl* stocks were balanced over *Kr:GFP-CyO*. *UAS-lgl^RNAi^* stocks were obtained from the Vienna *Drosophila* RNAi Center (lines # v51247 and v51249). *UAS miR-9a* and *miR-9a^F80^* flies were provided by Fen-Biao Gao (University of Massachusetts Medical School, MA, USA) and Eric Lai (Sloan-Kettering Cancer Center, NY, USA). *UAS-let-7* was obtained from Laura Johnston (Columbia University, NY, USA). *en-GAL4*, *GMR-GAL4*, and *UAS-GFP* were obtained from the Bloomington Stock Center (http://flystocks.bio.indiana.edu/). A recombinant stock containing *UAS-lgl-RNAi^51247^* and *UAS-lgl-RNAi^51249^* (third chromosome) was generated and a stock made with *en-GAL4* (second chromosome).

### Adult wing and eye sample preparations

A recombinant stock containing *UAS-lgl-RNAi^51247^* and *UAS-lgl-RNAi^51249^* (third chromosome) was generated and a stock made with *en-GAL4* (second chromosome). Wings of *en-GAL4/+; UAS-lgl^RNAi^/+*, *en-GAL4/+; UAS-lgl^RNAi^/UAS-miR-9a*, *en-GAL4/+; UAS-lgl^RNAi^/miR-9a^F80^* were removed under a dissecting light microscope and mounted on standard glass slides. Slides were scored for defects and examples of phenotypes were imaged using an Olympus DP71 imaging camera on a Leica MZ6 microscope. For *en-GAL4/+; UAS-lgl^RNAi^/+*, we scored *n*=132 wings. For *en-GAL4/+; UAS-lgl^RNAi^/UAS-miR-9a*, *n*=186. For *en-GAL4/+; UAS-lgl^RNAi^/miR-9a^F80^*, *n*=98 wings. For *en-GAL4/+; +/+*, *n*=140 wings. For *en-GAL4/+; UAS-miR-9a/+*, *n*=77 wings. For *en-GAL4/+; miR-9a^F80^*, *n*=70 wings. For *en-GAL4, UAS-GFP; UAS-lgl^RNAi^/+*, *n*=32. For *en-GAL4, UAS-GFP/+,+*, *n*=50. Images were processed using Adobe Photoshop and the posterior wing region as a proportion of total wing area was measured using ImageJ. The posterior wing region was defined as wing area posterior to the longitudinal vein L4. ‘Freehand selection’ was used to capture pixel areas and ‘Measure’ was used to compute the areas. For quantifying posterior wing region ratios we used a subset of samples: for *en-GAL4/+; UAS-lgl^RNAi^/+*, we imaged and measured *n*=16 wings. For *en-GAL4/+; UAS-lgl^RNAi^/UAS-miR-9a*, *n*=20. For *en-GAL4/+; UAS-lgl^RNAi^/miR-9a^F80^*, *n*=20 wings. For *en-GAL4, UAS-GFP/+; UAS-lgl^RNAi^/+* we imaged *n*=28 wings. For statistics, measurements within genotypes were checked for normality using the ad.test() in R. Differences between genotypes were calculated using t.test() in R for parametric distributions and wilcox.test() for non-parametric distributions. All tests used default options.

For adult eyes, flies were collected and imaged in the first 1-2 days after eclosion. Images were acquired using an Olympus DP71 camera mounted on a Leica MZ6 microscope and processed with ImageJ and Adobe Photoshop. For all genotypes we imaged 10-20 randomly selected flies (males and females).

### Brain dissection and imaging

Homozygous larvae were selected against GFP expression under UV light with a Leica MZ8 microscope and washed 3 times in 1× PBS. Cephalic complexes, consisting of brain lobes, ventral ganglion, and eye imaginal discs, were dissected from larvae and suspended in a drop of 1× PBS for imaging. Brain images were obtained using an Olympus DP71 imaging camera mounted on a Leica MZ6 microscope and processed with Adobe Photoshop.

### RNA preparation, microarrays and RT-PCR validation

For miRNA analysis, cephalic complexes were dissected from 20 third instar larvae per genotype and were pooled to create each time point sample. Three samples were collected per time point and total RNA was immediately extracted following dissection with a miRVana RNA extraction kit to conserve small RNA according to manufacturer's protocols (Ambion, Austin, TX, USA). RNA was quantified and evaluated for integrity with a nanodrop spectrophotometer and denaturing agarose gels.

For miR microarray analysis, total RNA was shipped to Genosensor (Phoenix, AZ, USA) where it was subjected to quality testing, hybridized with fluorescent probes and washed over an array spotted with cDNA complementary to 147 published *Drosophila* miRNAs. Fluorescence was imaged with a GenePix 4000B microarray scanner and measured using GenePix Pro 5.0.0.49 software.

To validate microarray results, Real-Time PCR was performed on select miRNAs. 1 μg RNA was annealed to poly (A) linkers and reverse transcribed with a one-step cDNA Synthesis Kit (GenoSensor, Phoenix, AZ, USA) according to manufacturer's protocols. Real-Time SYBR green Master Mix was combined with amplified cDNA and validated Real Time primers for *Drosophila let-7*, *miR-9a*, *miR-210*, and *U6* (GenoSensor, Phoenix, AZ, USA). Real-Time amplification reactions were loaded into a 384-well plate and run on an ABI 7900 Real Time thermocycler with an initial denaturation of 15 min at 94°C, 30-45 cycles of denaturation at 94°C for 30 s, annealing at 59°C for 15 s, and elongation 72°C for 30 s. Raw data was processed using a common threshold value. Fold changes were calculated with the delta delta Ct method using U6 as a housekeeping gene.

For mRNA analysis, RNA was isolated from 20 cephalic complexes (brain lobes and eye discs). Samples were from: wild-type day 0 third instar larvae and day 4 *lgl^27S3/E2S31^* mutant third instar larvae. 1 µg of total RNA was used for template preparation as per manufacturer's instructions, and hybridized to an Affymetrics 2.0 microarray gene chip. Gene-chips were washed and stained in the Affymetrix Fluidics Station 400 and scanned using the Hewlett-Packard GeneArray Scanner G2500A.

### Cell culture

MCF10A cell lines were obtained from American Type Culture Collection (ATCC) and cultured in Dulbecco's modified Eagle medium/F12 (DMEM/F12) supplemented with 5% Horse Serum (Invitrogen), 10 μg/ml insulin, 100 ng/ml Cholera toxin (Sigma Aldrich), 20 ng/ml Epidermal Growth Factor, 1% Penicillin-Streptomycin (Cellgro), and 0.5 μg/ml Hydrocortisone. All cells were grown at 37°C in 5% CO_2_. They were recently authenticated (Russ et al., 2012) and checked for contamination.

### Viral shRNA transductions

MISSION shRNA lentiviral particles containing nontarget control shRNA or *HUGL1* shRNAs and packaging vectors were purchased from Sigma Aldrich (NM_004140, clones TRCN0000117137-141). For transduction, virus was added to MCF10A cells at a multiplicity of infection (MOI) range of 1 to 3 in the presence of 8 μg/ml hexadimethrine bromide (Sigma Aldrich) in culture medium. Transduced cells were selected using puromycin dihydrochloride (Sigma Aldrich) at 2 μg/ml. Stable lines were used as heterogenous populations; clones were not selected.

### Real time PCR array

Total RNA was isolated from the MCF10A control and *HUGL1* shRNA cell lines two weeks after transduction using the RNeasy kit from Qiagen (Valencia, CA, USA). Total RNA concentration and purity in the eluted samples was tested using a NanoDrop spectrophotometer (NanoDrop Technologies). RNA quality was examined in 1% denaturing agarose gels and sharp bands at 18S and 28S ribosomal were verified. Following RNA preparation, the samples were treated with DNase using the RNase-Free DNase Set (Qiagen) to ensure elimination of genomic DNA, and the extracted RNA was converted to cDNA using the RT^2^ First Strand Kit (Qiagen) following manufacturer's instructions. cDNA was stored at −20°C until used for gene expression profiling.

The RT^2^ Profiler PCR Array System (SABiosciences, Qiagen) was used to evaluate the cell lines for differential gene expression. The genes evaluated in this Breast Cancer RT PCR array include multiple genes involved in breast cancer carcinogenesis. SYBR based Real-time PCR detection was carried out per the manufacturer's instructions. The array was cycled on an ABI 7900HT real-time cycler on the following program: 1 cycle of 10 min at 95°C followed by 40 cycles of 15 s at 95°C and 1 min at 60°C. Raw data were processed in ABI software using similar baseline and threshold values and exported to a template Excel file (Microsoft) for analysis. Analyses of the raw C_T_ values were conducted using the ΔΔC_T_ method through the SABiosciences Data Analysis Web Portal (www.sabiosciences.com). Runs that did not pass quality control tests were eliminated and four replicates of each treatment were used for statistical analysis.

### Western blots

Cultured cells were lysed in ice-cold lysis buffer containing 20 mM TRIS pH 7.5, 150 mM NaCl, 1% NP40, 5 mM EDTA pH 8.0, 1% NaF, 1% NaVO3, 0.1% NH_4_ Molybdate and 8% Complete phosphatase and protease inhibitor (Roche). The lysates were centrifuged at 13,000 rpm for 10 min at 4°C and supernatant was collected for western blot analysis. 20μg protein lysate was separated by SDS-PAGE (7%) and transferred to PVDF membrane (Millipore). The membrane was blocked in 5% milk in PBS/0.1% Tween solution and then used for immunoblotting. The primary antibodies, anti-HUG1 (911-1010, cat # H00003996-M01) and anti-β-actin (AC-74) were purchased from Abnova and Sigma, respectively and the secondary antibody, conjugated to horseradish peroxidase (HRP), goat anti-mouse IgG HRP was purchased from Invitrogen/Molecular Probes. Proteins on the membrane were treated with Super Signal Chemiluminescent Substrate (Pierce), visualized on Imagetech-B film (American X-ray) and developed with a Konica SRX-101C.

### Microarray analysis, microRNA target matching and GO-term analysis

Both microarrays (microRNA and mRNA) were analysed using the Bioconductor package in the R statistical software environment. For microRNAs, normalization was done between arrays and correlation was determined for technical replicates. Normalization was done for arrays on a comparison group basis (i.e. for the day 3 lgl^1^/lgl^U334^ compared to day 0 wild-type only those two groups were normalized rather than all). For mRNAs, background correction and normalization was done using the robust-multichip-array (rma) algorithm (Parrish and Spencer, 2004). Data for both experiments were then fitted to separate linear models using the limma package (Wettenhall and Smyth, 2004), which calculated fold changes and *P*-values (using Benjamini-Hochberg multiple testing correction).

Computationally determined targets with good mirSVR scores and conservation across species were downloaded from microRNA.org. This list was filtered for the miRNAs of interest and the predicted targets were matched to mRNAs of interest. Upregulated miRNAs were matched only to downregulated mRNAs and vice versa. The targeting network was visualized using Cytoscape software version 2.8.3 (Shannon et al., 2003). The Bingo plug-in (version 2.44) for Cytoscape was then used to compute enrichment for GO-terms of the miRNAs and mRNAs together (Maere et al., 2005). The background used for enrichment tests consisted of all miRNAs and predicted targets of the same aforementioned list from microRNA.org. Fisher exact tests were used with Benjamini-Hochberg multiple testing correction to determine if groups of miRNAs/mRNAs were significantly associated with a specific GO-term. Only those with a corrected *P*-value of 0.05 or less were included in the results.

### Wing imaginal disc immunostaining and quantification

Wing discs were dissected from wandering third instar larve in Grace's medium (source) and incubated for 1 h to incorporate a BrDU analog, EdU, using the Click-iT Kit (Invitrogen). Discs were fixed in 3.5% formaldehyde in PBS, pH 7.2, permeabilized with 0.1% Triton, blocked with 3% BSA, and labeled. The presence of cleaved caspase was detected using Dcp-1 Antibody (Cell Signaling, #9578) at 1/500 dilution, and detected with anti-rabbit Alexa-647 at 1/750. Anti-GFP-FITC (Rockland, #600-402-215) was used at 1/200 dilution and Hoechst 33344 (Life Technologies) at 1/10,000. Discs were mounted in 4% n-propyl gallate in glycerol and imaged on a Zeiss 510 Meta Confocal Microscope. Images were processed by Adobe Photoshop. Total wing disc and the engrailed domain (GFP positive) were defined with the ‘magnetic lasso’ tool. For the measurements shown in Fig. S1E, to reduce background noise, only the top-most epithelial layer corresponding to the wing pouch was measured. For statistics, measurements within genotypes were checked for normality using the shapiro.test() in R. Differences between genotypes were calculated using t.test() in R. All tests used default options.

### Statistical analyses

Statistical analyses are as described for individual methods.

## Supplementary Material

Supplementary information
