# Supplementary material for: miR-9a mediates the role of Lethal giant larvae as an epithelial growth inhibitor in Drosophila
Source: Biol Open. 2017 Dec 20;7(1):bio027391. doi: 10.1242/bio.027391 (PMC5829493; doi:10.1242/bio.027391)
Supplement: Supplementary information [file biolopen-7-027391-s1.pdf]

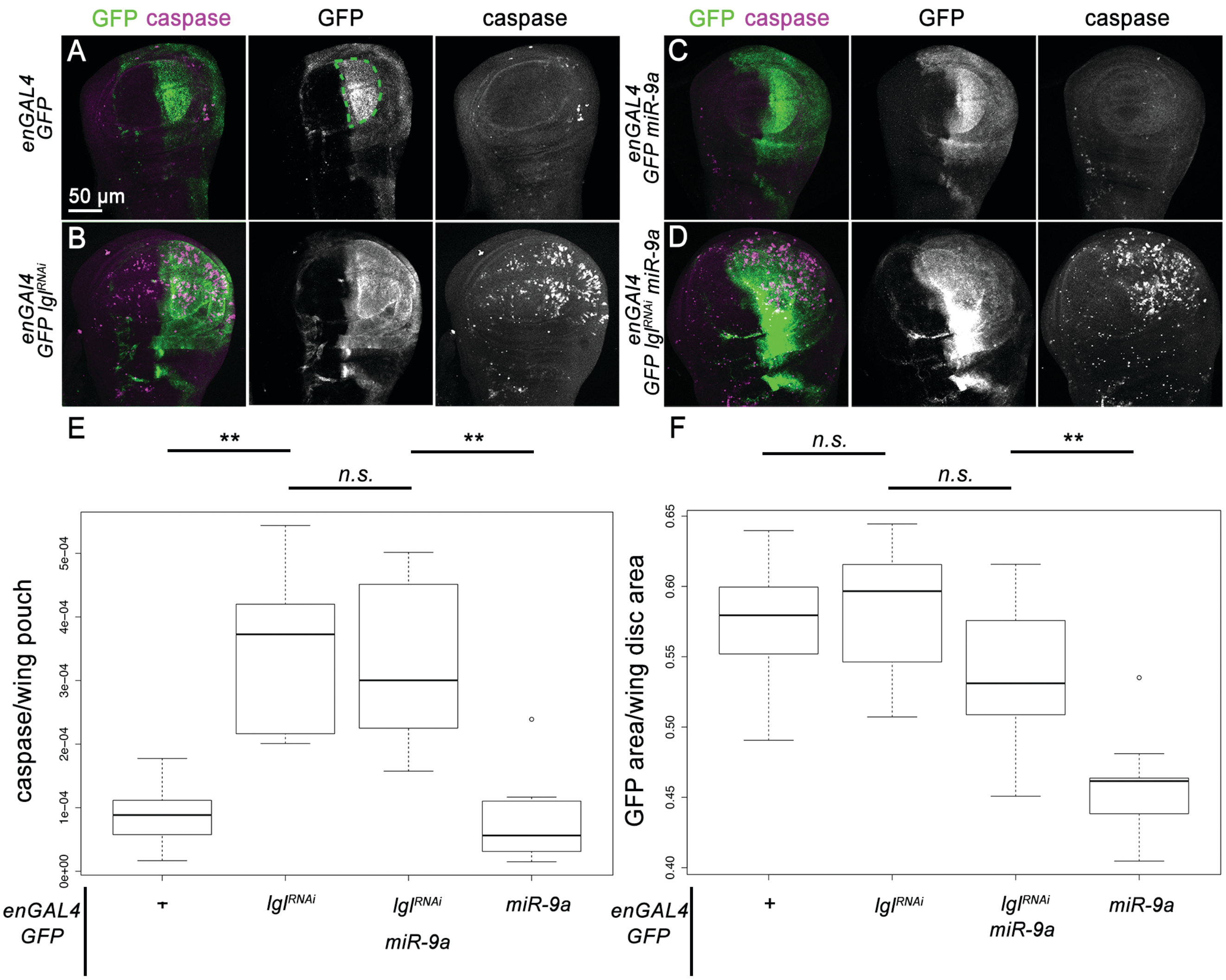

Supplemental Figure S1 – miR-9a does not significantly affect growth or apoptosis in third instar wing imaginal discs.

(A – D) Third instar wing imaginal discs immunostained, as indicated. Genotypes, as shown. Dashed area in panel A, GFP channel indicates the wing pouch area within the engrailed domain that was used to quantify caspase activity in (E). (E) Caspase activity in the wing pouch area. (F) Size of engrailed domain (GFP) normalized to whole wing disc. * - P_{value} <0.05, ** - P_{value} <0.01, *** - P_{value} <0.001. n.s. – not significant.

Biology Open • Supplementary information

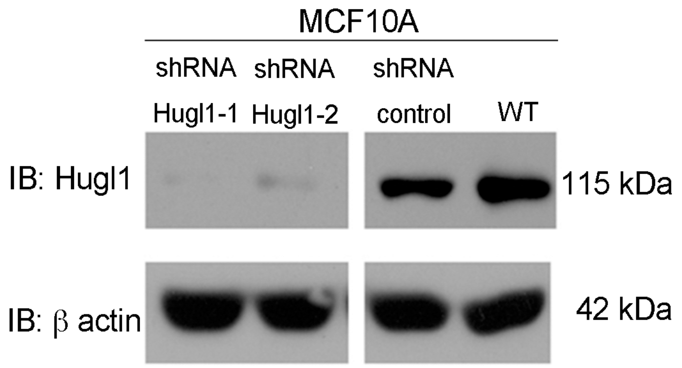

Supplemental Figure S2 - Hugl1 knock-down in human breast epithelial cells.

Stable knock-down was established in MCF10As with transduction of *HUGL1* or control shRNA lentiviral particles and selected with puromycin. Protein lysates were isolated from cell lines, 20 µg of protein were separated by SDS PAGE and analyzed by immunoblot using the antibodies: anti-HUGL1 (911–1010, cat # H00003996-M01, Abnova, 1/1,000) and anti-β actin (loading control). Secondary, HRP conjugated antibodies were used at 1/10,000 (Invitrogen). Molecular weights as shown.

Table S1: Dysregulated miRNAs in 0, 3, and 5 day *lgl* mutants

0 day lgl^{1}/lgl^{U334} compared to 0 day $P[lgl^{+}];lgl^{1}/lgl^{U334}$

ID	logFC	AveExpr	t	P.Value	adj.P.Val	B
**dme-let-7**	-1.1952	7.8836	-19.0271	1.18E-13	2.71E-11	21.5407
**dme-miR-210**	-1.4933	8.6508	-10.6996	2.19E-09	2.50E-07	11.5605
**dme-miR-34**	1.1900	8.4691	9.8950	7.61E-09	5.81E-07	10.2787
**dme-miR-125**	-0.4540	7.3833	-9.6771	1.08E-08	6.19E-07	9.9188
**dme-miR-100**	-0.4843	7.4360	-8.7235	5.34E-08	2.45E-06	8.2755
**dme-miR-277**	0.6403	8.4971	8.3621	1.01E-07	3.85E-06	7.6226
**dme-miR-11**	-0.5808	10.3621	-8.1093	1.59E-07	5.19E-06	7.1559
**dme-miR-1010**	-0.6484	8.6646	-7.1498	9.61E-07	2.75E-05	5.3064
**dme-miR-965**	-0.4585	7.9763	-6.6570	2.54E-06	6.47E-05	4.3087
**dme-miR-317**	0.5697	10.3094	5.7691	1.59E-05	3.65E-04	2.4334
**dme-miR-993**	-0.2770	7.9174	-5.6604	2.01E-05	4.18E-04	2.1972
**dme-miR-279**	-0.5175	10.6893	-5.4195	3.37E-05	6.43E-04	1.6701
**dme-miR-14**	1.2090	10.1930	5.2976	4.39E-05	7.73E-04	1.4012
**dme-miR-927**	-0.3746	8.0153	-5.1404	6.19E-05	1.01E-03	1.0525
**dme-miR-275**	-0.6774	8.5163	-5.1011	6.75E-05	1.03E-03	0.9650
**dme-miR-34-pre**	0.2323	7.4936	4.8206	1.25E-04	1.80E-03	0.3373
**dme-miR-999**	-0.5340	8.4509	-4.7458	1.48E-04	2.00E-03	0.1692
**dme-miR-1012**	-0.2284	7.5795	-4.7126	1.60E-04	2.03E-03	0.0945
**dme-miR-133**	0.5622	8.2516	4.2154	4.87E-04	5.88E-03	-1.0285
**dme-miR-9a**	-0.6381	12.4448	-4.1853	5.22E-04	5.97E-03	-1.0965
**dme-miR-980**	0.3377	7.5321	3.9963	8.00E-04	8.72E-03	-1.5235
**dme-miR-289**	0.3944	7.7972	3.9165	9.58E-04	9.97E-03	-1.7032
**dme-miR-278**	0.3375	7.8889	3.8583	1.09E-03	1.09E-02	-1.8343
**dme-miR-iab-4-pre**	-0.1231	7.5461	-3.8222	1.19E-03	1.13E-02	-1.9154
**dme-miR-9b**	-0.3932	9.8743	-3.7644	1.35E-03	1.24E-02	-2.0451
**dme-miR-1004**	0.2648	7.7865	3.6919	1.59E-03	1.40E-02	-2.2074
**dme-miR-31a**	0.2381	8.4021	3.6274	1.84E-03	1.51E-02	-2.3513
**dme-miR-14-pre**	0.1649	7.5567	3.6258	1.85E-03	1.51E-02	-2.3549
**dme-miR-276a**	-0.7282	13.1731	-3.5503	2.19E-03	1.73E-02	-2.5229
**dme-miR-2b**	0.4380	13.0940	3.4440	2.78E-03	2.13E-02	-2.7584
**dme-miR-966**	0.1820	7.7112	3.3724	3.27E-03	2.42E-02	-2.9160
**dme-miR-276b**	-0.7424	13.0213	-3.2932	3.90E-03	2.79E-02	-3.0895
**dme-miR-1**	0.3115	10.8684	3.2351	4.45E-03	3.04E-02	-3.2161
**dme-miR-277-pre**	-0.1384	7.2567	-3.2289	4.51E-03	3.04E-02	-3.2296
**dme-miR-281**	0.1770	7.3492	3.1949	4.86E-03	3.18E-02	-3.3032
**dme-miR-995**	0.4377	9.7921	3.0419	6.82E-03	4.26E-02	-3.6322
**dme-miR-1003**	-0.1830	7.5222	-3.0380	6.88E-03	4.26E-02	-3.6404
**dme-miR-958**	0.2199	7.5380	2.9535	0.0082866	0.04993776	-3.8198

Biology Open • Supplementary information

3 day *lgl*^{1}/*lgl*^{U334} compared to 0 day *P[lgl*$^{+}$*];lgl*^{1}/*lgl*^{U334}

ID	logFC	AveExpr	t	P.Value	adj.P.Val	B
**dme-let-7**	-1.0533	8.3673	-14.9044	2.67E-12	6.12E-10	18.2895
**dme-miR-980**	0.8383	7.9187	12.5812	5.80E-11	6.65E-09	15.1453
**dme-miR-9a**	-0.8635	13.1862	-9.5281	7.03E-09	5.16E-07	10.2232
**dme-miR-210**	-0.9062	9.3872	-9.3865	9.01E-09	5.16E-07	9.9692
**dme-miR-317**	0.9628	11.0489	8.5210	4.31E-08	1.97E-06	8.3612
**dme-miR-263b**	-0.5700	9.6784	-7.5657	2.72E-07	1.04E-05	6.4723
**dme-miR-275**	-0.7146	8.9456	-7.4275	3.58E-07	1.17E-05	6.1891
**dme-miR-34**	0.5519	8.5042	6.6220	1.89E-06	5.40E-05	4.4879
**dme-miR-13a**	0.6062	12.6264	6.5192	2.35E-06	5.97E-05	4.2649
**dme-miR-993**	-0.4707	8.2093	-6.1270	5.47E-06	1.25E-04	3.4020
**dme-miR-125**	-0.4108	7.6189	-5.8409	1.03E-05	2.13E-04	2.7614
**dme-miR-33**	0.4658	9.1368	5.6732	1.49E-05	2.84E-04	2.3820
**dme-miR-100**	-0.4608	7.6932	-5.4932	2.23E-05	3.93E-04	1.9717
**dme-miR-278-pre**	-0.3120	7.1975	-5.2919	3.52E-05	5.75E-04	1.5094
**dme-miR-184-pre**	0.6371	9.3179	5.2305	4.04E-05	6.18E-04	1.3680
**dme-miR-1**	-0.5427	11.0650	-4.8038	1.08E-04	1.54E-03	0.3775
**dme-miR-263a**	-0.5575	10.1408	-4.6481	1.55E-04	2.08E-03	0.0141
**dme-miR-10**	-0.4222	7.9903	-4.5448	1.97E-04	2.50E-03	-0.2273
**dme-miR-307**	-0.4322	10.6377	-4.5197	2.09E-04	2.51E-03	-0.2861
**dme-miR-284**	-0.2277	7.5347	-4.3609	3.02E-04	3.46E-03	-0.6575
**dme-miR-2c**	0.4062	13.3650	3.7620	1.23E-03	1.34E-02	-2.0506
**dme-miR-13b-1-pre**	0.2109	7.4914	3.7311	1.32E-03	1.37E-02	-2.1216
**dme-miR-6**	-0.2170	7.3993	-3.6767	1.49E-03	1.49E-02	-2.2467
**dme-miR-306-pre**	-0.2582	12.6311	-3.5809	1.87E-03	1.78E-02	-2.4662
**dme-miR-276***	-0.5175	11.2024	-3.3771	2.99E-03	2.72E-02	-2.9284
**dme-bantam**	0.5755	12.0156	3.3641	3.08E-03	2.72E-02	-2.9576
**dme-miR-308**	0.3263	7.7307	3.2097	4.39E-03	3.73E-02	-3.3023
**dme-miR-304**	0.3292	7.6465	3.1671	4.84E-03	3.96E-02	-3.3966
**dme-miR-277**	0.2987	8.7257	3.0740	5.98E-03	4.67E-02	-3.6011
**dme-miR-305-pre**	-0.1593	7.5443	-3.0642	6.12E-03	4.67E-02	-3.6227
**dme-miR-1004**	-0.2661	7.7498	-3.0424	6.43E-03	4.75E-02	-3.6700
**dme-miR-281**	0.1616	7.4236	3.0140	6.85E-03	4.90E-02	-3.7319

5 day *lgl*^{1}*/lgl*^{U334} compared to 0 day *P[lgl*$^{+}$*];lgl*^{1}*/lgl*^{U334}

ID	logFC	AveExpr	t	P.Value	adj.P.Val	B
**dme-miR-2c**	1.0282	14.0254	13.2719	3.64E-11	8.35E-09	15.6930
**dme-miR-304**	0.7046	7.9413	12.0993	1.81E-10	1.71E-08	14.0522
**dme-miR-13a**	0.8625	13.1794	11.9516	2.24E-10	1.71E-08	13.8363
**dme-miR-317**	1.0370	11.3534	11.1805	6.96E-10	3.98E-08	12.6744
**dme-miR-993**	-0.5957	8.3707	-9.1374	1.90E-08	8.69E-07	9.2797
**dme-miR-263b**	-0.6943	9.8754	-8.4092	6.95E-08	2.65E-06	7.9472
**dme-miR-2b**	0.5961	14.4505	8.0737	1.29E-07	4.23E-06	7.3098
**dme-miR-999**	0.7691	9.7441	7.9269	1.70E-07	4.28E-06	7.0263
**dme-miR-282**	0.6003	8.1291	7.9135	1.75E-07	4.28E-06	7.0004
**dme-miR-6**	-0.3061	7.4670	-7.8784	1.87E-07	4.28E-06	6.9320
**dme-miR-252**	-0.8144	12.3615	-7.8128	2.12E-07	4.40E-06	6.8040
**dme-miR-263a**	-0.7836	10.3156	-7.6814	2.72E-07	5.19E-06	6.5457
**dme-miR-10**	-0.5001	8.1041	-7.1299	8.02E-07	1.41E-05	5.4368
**dme-miR-1**	-0.9589	11.1261	-7.0625	9.18E-07	1.50E-05	5.2984
**dme-miR-276a-pre**	0.6169	8.7686	6.8866	1.31E-06	1.95E-05	4.9346
**dme-miR-965**	0.4931	9.0821	6.8664	1.36E-06	1.95E-05	4.8926
**dme-bantam**	1.0867	12.6680	6.6653	2.06E-06	2.77E-05	4.4712
**dme-miR-980**	0.4596	7.8368	6.5769	2.47E-06	3.15E-05	4.2844
**dme-miR-190**	-0.8557	9.4161	-6.4386	3.30E-06	3.97E-05	3.9901
**dme-miR-1016**	-0.2948	7.4521	-6.3923	3.63E-06	4.06E-05	3.8908
**dme-miR-961**	0.3011	7.4331	6.3806	3.72E-06	4.06E-05	3.8657
**dme-miR-278-pre**	-0.3539	7.2622	-6.2988	4.42E-06	4.60E-05	3.6899
**dme-miR-87**	-0.4176	7.8336	-6.2184	5.24E-06	5.22E-05	3.5164
**dme-miR-1008**	0.3915	7.7884	6.1801	5.69E-06	5.43E-05	3.4335
**dme-miR-284**	-0.2806	7.6264	-6.0624	7.31E-06	6.36E-05	3.1774
**dme-miR-981**	0.4709	8.6243	6.0563	7.40E-06	6.36E-05	3.1641
**dme-miR-9a**	-0.7119	13.6559	-6.0504	7.50E-06	6.36E-05	3.1512
**dme-miR-31b**	-0.4554	8.6695	-6.0328	7.79E-06	6.37E-05	3.1128
**dme-miR-1004**	-0.4269	7.7978	-5.7614	1.40E-05	1.10E-04	2.5156
**dme-miR-315**	1.0454	12.0901	5.7432	1.46E-05	1.11E-04	2.4752
**dme-miR-2a**	-0.8350	13.3461	-5.5134	2.41E-05	1.78E-04	1.9631
**dme-miR-iab-4-pre**	-0.2641	7.7916	-5.3719	3.29E-05	2.32E-04	1.6451
**dme-miR-1001**	0.3061	7.3204	5.3654	3.34E-05	2.32E-04	1.6306
**dme-miR-304-pre**	0.2754	7.4632	5.2469	4.35E-05	2.93E-04	1.3630
**dme-miR-13a-pre**	0.2612	7.4215	5.0880	6.21E-05	4.06E-04	1.0023
**dme-miR-963**	0.2913	7.4617	5.0287	7.09E-05	4.51E-04	0.8675
**dme-miR-2c-pre**	0.3680	7.5646	5.0032	7.51E-05	4.65E-04	0.8093
**dme-miR-31a**	-0.3609	8.7439	-4.9400	8.67E-05	5.22E-04	0.6650

Biology Open • Supplementary information

ID	logFC	AveExpr	t	P.Value	adj.P.Val	B
**dme-miR-33**	0.3360	9.2948	4.9064	9.35E-05	5.49E-04	0.5884
**dme-miR-316**	-0.3280	9.1401	-4.7003	1.49E-04	8.55E-04	0.1162
**ID**	**logFC**	**AveExpr**	**t**	**P.Value**	**adj.P.Val**	**B**
**dme-miR-275**	-0.4241	9.2926	-4.6328	1.74E-04	9.71E-04	-0.0388
**dme-miR-1007**	0.2836	7.5222	4.6231	1.78E-04	9.71E-04	-0.0610
**dme-miR-1017**	-0.1644	7.5899	-4.5642	2.04E-04	1.08E-03	-0.1964
**dme-miR-998**	0.4655	11.1510	4.5449	2.13E-04	1.11E-03	-0.2408
**dme-miR-966**	0.3233	8.1191	4.5308	2.20E-04	1.12E-03	-0.2731
**dme-miR-306**	-0.3442	12.9731	-4.4626	2.57E-04	1.28E-03	-0.4300
**dme-miR-184***	-0.1825	7.6669	-4.3548	3.29E-04	1.60E-03	-0.6781
**dme-miR-1003**	0.4171	8.1739	4.3178	3.58E-04	1.71E-03	-0.7632
**dme-miR-276a**	-0.4471	14.5395	-4.3014	3.72E-04	1.74E-03	-0.8009
**dme-miR-9a-pre**	-0.1806	7.4248	-4.2607	4.09E-04	1.87E-03	-0.8945
**dme-miR-210**	-0.4728	9.8374	-4.1458	5.32E-04	2.39E-03	-1.1586
**dme-miR-995**	0.7444	10.6417	4.0744	6.27E-04	2.76E-03	-1.3226
**dme-miR-276***	-0.7684	11.4119	-4.0345	6.88E-04	2.97E-03	-1.4142
**dme-miR-1009**	0.1635	7.3192	3.8127	1.15E-03	4.86E-03	-1.9214
**dme-miR-263b-pre**	-0.1575	7.0955	-3.7354	1.37E-03	5.70E-03	-2.0974
**dme-miR-314**	0.4129	7.5035	3.7109	1.45E-03	5.92E-03	-2.1529
**dme-miR-958**	-0.3141	7.5279	-3.5808	1.95E-03	7.84E-03	-2.4472
**dme-let-7**	-0.3520	8.9411	-3.5142	2.27E-03	8.97E-03	-2.5972
**dme-miR-957**	0.3031	8.6398	3.5022	2.34E-03	9.06E-03	-2.6241
**dme-miR-316-pre**	-0.4749	8.1542	-3.4446	2.66E-03	1.02E-02	-2.7531
**dme-miR-317-pre**	0.2079	7.4198	3.4353	2.72E-03	1.02E-02	-2.7739
**dme-miR-iab-4-5p**	-0.1951	7.8152	-3.3753	3.12E-03	1.15E-02	-2.9078
**dme-miR-13b-1-pre**	0.1667	7.5634	3.2549	4.10E-03	1.49E-02	-3.1744
**dme-miR-276b**	-0.3918	14.3933	-3.1978	4.66E-03	1.67E-02	-3.2997
**dme-miR-11**	-0.3825	11.3233	-3.1613	5.06E-03	1.78E-02	-3.3797
**dme-miR-956**	0.1213	7.2064	3.1375	5.34E-03	1.85E-02	-3.4315
**dme-miR-279**	0.2874	12.1330	2.9846	7.52E-03	2.57E-02	-3.7618
**dme-miR-100**	-0.2469	7.9272	-2.9332	8.42E-03	2.84E-02	-3.8715
**dme-miR-280-pre**	0.1397	7.3562	2.9265	8.55E-03	2.84E-02	-3.8857
**dme-miR-184-pre**	0.3575	9.3942	2.9186	8.70E-03	2.85E-02	-3.9025
**dme-miR-12-pre**	0.2203	7.6314	2.9056	8.96E-03	2.86E-02	-3.9302
**dme-miR-307**	-0.2688	10.9166	-2.9039	8.99E-03	2.86E-02	-3.9337
**dme-miR-970**	0.3674	9.6759	2.8757	9.57E-03	3.00E-02	-3.9934
**dme-miR-284-pre**	-0.1752	7.2424	-2.8265	1.07E-02	3.30E-02	-4.0970
**dme-miR-184**	-0.2550	10.9838	-2.6302	1.63E-02	4.99E-02	-4.5021

Table S2: Dysregulated mRNAs in *lgl* mutants*

*There are multiple Affy IDs for 4 mRNAs so all were included for completeness

Affy ID	Entrez Gene ID	Gene Symbol	log_2FC	Average Expression	t	P-Value	Adjusted P-Value	B
**1627736_at**	43826	Actbeta	3.7702	5.5554	43.8662	3.91E-07	5.81E-03	5.6847
**1634988_a_at**	38946	CG17352	3.2902	6.0563	38.8127	6.79E-07	5.81E-03	5.5138
**1639643_at**	33483	CG18557	2.3836	5.1025	30.4076	2.05E-06	8.81E-03	5.0720
**1639442_a_at**	32821	Tsf1	1.8542	7.8999	29.5604	2.33E-06	8.81E-03	5.0113
**1629220_at**	39150	Ilp2	-2.3716	8.2999	-26.0565	4.11E-06	1.12E-02	4.7149
**1626642_at**	38994	CG6486	2.6329	9.2012	26.0406	4.12E-06	1.12E-02	4.7134
**1632119_s_at**	35940	ltd	-1.6938	5.8817	-24.1769	5.76E-06	1.36E-02	4.5189
**1638131_s_at**	37196	5-HT1A	-2.3559	4.9898	-22.2862	8.30E-06	1.55E-02	4.2887
**1624269_at**	33530	gkt	2.8271	6.7741	21.3542	1.01E-05	1.55E-02	4.1609
**1638305_at**	39933	Mip	-1.6650	6.7060	-20.9997	1.08E-05	1.55E-02	4.1096
**1627825_at**	38992	CG13305	1.9988	7.6891	20.7531	1.14E-05	1.55E-02	4.0729
**1625850_at**	33583	odd	1.2544	5.6890	19.7069	1.44E-05	1.70E-02	3.9079
**1629944_at**	41246	CG12814	1.9773	4.7786	19.6531	1.46E-05	1.70E-02	3.8990
**1632688_s_at**	38473	CG11594	-1.2165	5.2039	-19.2403	1.61E-05	1.70E-02	3.8292
**1626439_at**	50191	CG15353	-2.9632	9.4435	-19.2108	1.62E-05	1.70E-02	3.8241
**1635273_s_at**	32930	kek5	3.9559	6.6035	18.3824	1.97E-05	1.97E-02	3.6754
**1625442_a_at**	45928	shi	-1.0590	7.3075	-18.1630	2.08E-05	1.97E-02	3.6341
**1635522_a_at**	34024	santa-maria	-1.5899	7.8036	-16.7758	2.97E-05	2.13E-02	3.3520
**1624634_at**	5740633	nvd	-2.4472	3.6175	-16.4243	3.26E-05	2.13E-02	3.2744
**1638601_at**	5740359	spok	-5.9969	5.4397	-16.3771	3.31E-05	2.13E-02	3.2638
**1634428_at**	34307	CG5924	1.0170	6.6738	16.3095	3.37E-05	2.13E-02	3.2485
**1641490_s_at**	33941	Tsp	1.0532	5.8882	16.2218	3.45E-05	2.13E-02	3.2284
**1635665_at**	35573	Tdc1	3.2044	6.0324	15.8974	3.78E-05	2.13E-02	3.1529
**1627783_at**	42191	CG18599	1.5257	6.1510	15.8807	3.79E-05	2.13E-02	3.1490
**1630457_s_at**	41144	by	1.1613	5.3217	15.7307	3.96E-05	2.13E-02	3.1132
**1632430_at**	32821	Tsf1	1.7148	9.4151	15.5856	4.13E-05	2.13E-02	3.0780
**1630642_at**	33994	Pvf2	1.8023	4.8666	15.5659	4.15E-05	2.13E-02	3.0732
**1637410_s_at**	33156	l(2)gl	-4.8783	4.3103	-15.5398	4.18E-05	2.13E-02	3.0668
**1629569_at**	32378	Fbxl4	2.9784	5.1256	15.5157	4.21E-05	2.13E-02	3.0609
**1633880_s_at**	40157	Ir76a	1.6836	5.8404	15.5000	4.23E-05	2.13E-02	3.0571
**1632097_at**	32501	CG15646	1.7923	7.6613	15.4636	4.27E-05	2.13E-02	3.0481
**1625473_at**	41935	CG4221	1.4136	4.9077	15.1260	4.72E-05	2.19E-02	2.9633
**1630683_at**	42058	Patr-1	2.0026	6.9114	15.1081	4.74E-05	2.19E-02	2.9587
**1635283_at**	260645	nimB2	1.2054	6.2397	15.0646	4.80E-05	2.19E-02	2.9475
**1634573_a_at**	37038	grh	1.8795	8.5670	14.9710	4.94E-05	2.19E-02	2.9234
**1632212_at**	35358	CG14401	1.6569	4.8895	14.8499	5.12E-05	2.19E-02	2.8918
**1632860_at**	38508	Cpr64Aa	6.2574	7.8120	14.8242	5.16E-05	2.19E-02	2.8850

Biology Open • Supplementary information

**1641548_at**	38714	CG10289	-2.6185	6.8741	-14.7921	5.21E-05	2.19E-02	2.8765
**1639741_at**	43158	HLHm5	-2.1675	5.6872	-14.5223	5.65E-05	2.29E-02	2.8042
**1639177_at**	3346202	IFa	-1.4948	7.7829	-14.4957	5.70E-05	2.29E-02	2.7970
**1631730_at**	36081	CG12911	2.1987	4.2267	14.4341	5.81E-05	2.29E-02	2.7802
**1636275_a_at**	3346192	Vmat	-1.6016	4.5092	-14.2162	6.22E-05	2.31E-02	2.7198
**1625195_s_at**	36171	shn	-1.5790	6.1515	-14.1516	6.34E-05	2.31E-02	2.7016
**1641344_a_at**	44018	cas	1.8185	9.3309	14.0391	6.57E-05	2.32E-02	2.6697
**1625197_at**	40680	exba	-1.2741	9.2761	-14.0214	6.61E-05	2.32E-02	2.6646
**1641423_at**	34037	CG6739	3.0252	6.3249	13.9192	6.83E-05	2.33E-02	2.6352
**1636242_at**	31220	Ilp6	-1.7116	5.7638	-13.8167	7.06E-05	2.33E-02	2.6054
**1638984_s_at**	40928	CG17816	-2.0394	6.1928	-13.7355	7.24E-05	2.33E-02	2.5816
**1623612_at**	36163	Spn47C	3.7865	5.7263	13.3169	8.31E-05	2.37E-02	2.4554
**1625570_at**	38510	Cpr64Ac	2.0393	5.9655	13.2671	8.45E-05	2.37E-02	2.4400
**1625325_s_at**	39212	simj	2.1170	6.8777	13.2392	8.53E-05	2.37E-02	2.4314
**1634364_s_at**	39518	stv	1.6982	7.0937	13.2374	8.53E-05	2.37E-02	2.4308
**1628884_at**	32099	PGRP-SA	3.6735	6.4673	13.1212	8.87E-05	2.37E-02	2.3945
**1624344_at**	37479	CG17922	-1.0538	5.8498	-13.0763	9.01E-05	2.37E-02	2.3803
**1630476_s_at**	37641	nahoda	2.7278	5.8647	13.0763	9.01E-05	2.37E-02	2.3803
**1627214_s_at**	39694	CG7650	-1.0387	5.7735	-13.0562	9.07E-05	2.37E-02	2.3740
**1636991_s_at**	37999	emp	1.2611	7.1208	13.0320	9.15E-05	2.37E-02	2.3663
**1638724_at**	34775	CG18507	1.5093	6.2276	12.9891	9.28E-05	2.37E-02	2.3526
**1624663_a_at**	36372	vis	-2.0836	3.3581	-12.9561	9.39E-05	2.37E-02	2.3421
**1634306_at**	43310	Klp98A	1.1438	7.2251	12.9474	9.41E-05	2.37E-02	2.3393
**1639320_a_at**	35190	Ddc	-1.1548	6.3642	-12.9059	9.55E-05	2.37E-02	2.3260
**1640586_at**	32037	CG1537	-1.1285	7.6745	-12.8774	9.64E-05	2.37E-02	2.3168
**1639181_at**	40893	CG14598	-2.0127	4.4031	-12.8365	9.78E-05	2.37E-02	2.3035
**1624362_at**	50190	Nplp4	-3.1264	6.5352	-12.8045	9.89E-05	2.37E-02	2.2931
**1635787_at**	33676	CG15630	-1.6754	6.0828	-12.5339	1.09E-04	2.49E-02	2.2037
**1633852_at**	32245	fne	-1.4616	9.1095	-12.5001	1.10E-04	2.49E-02	2.1924
**1630860_at**	38469	scrt	-1.1794	7.4293	-12.4939	1.10E-04	2.49E-02	2.1903
**1640465_at**	34485	CG17124	-1.5246	9.6502	-12.3935	1.14E-04	2.49E-02	2.1563
**1630065_at**	41820	CG6912	1.6674	6.0359	12.0900	1.27E-04	2.73E-02	2.0512
**1641499_at**	41739	CG3259	1.3001	4.3453	12.0542	1.29E-04	2.73E-02	2.0386
**1630186_at**	35911	CG13743	-1.5346	5.4803	-12.0393	1.30E-04	2.73E-02	2.0333
**1634507_s_at**	35212	CG17549	1.9848	7.5373	11.8850	1.37E-04	2.78E-02	1.9782
**1640057_at**	38134	CG9192	2.4181	5.4043	11.7409	1.45E-04	2.78E-02	1.9259
**1632339_s_at**	42066	cher	1.7899	7.4597	11.7186	1.46E-04	2.78E-02	1.9178
**1624501_at**	31226	CG12496	1.5345	5.4483	11.7077	1.47E-04	2.78E-02	1.9137
**1634302_s_at**	43444	CG14516	-1.7385	5.6462	-11.6742	1.49E-04	2.78E-02	1.9014
**1640390_at**	37626	CG3649	1.8912	4.2033	11.6308	1.51E-04	2.78E-02	1.8854
**1628732_at**	31394	pon	1.0409	9.0410	11.6103	1.52E-04	2.78E-02	1.8778
**1634957_at**	41170	Dh	-2.5777	6.8927	-11.6084	1.53E-04	2.78E-02	1.8771
**1626857_at**	42762	CG4408	-2.6816	5.9143	-11.6070	1.53E-04	2.78E-02	1.8765

**1626405_at**	36290	Drep-1	-1.3358	5.6153	-11.5839	1.54E-04	2.78E-02	1.8680
**1633793_at**	38801	unc-13-4A	-1.3103	4.4416	-11.4373	1.63E-04	2.87E-02	1.8129
**1626837_a_at**	38562	CG42540	-1.3312	7.5787	-11.4280	1.63E-04	2.87E-02	1.8094
**1626109_a_at**	36236	Drip	2.2568	5.2705	11.2605	1.74E-04	2.96E-02	1.7452
**1629981_at**	36615	LamC	1.5945	7.3958	11.2586	1.75E-04	2.96E-02	1.7445
**1636804_at**	31055	CG14629	1.3724	6.7000	11.2035	1.78E-04	2.96E-02	1.7231
**1624125_at**	33627	ft	1.6222	8.2749	11.1552	1.82E-04	2.96E-02	1.7043
**1631604_at**	33894	CG42369	2.5174	4.6944	11.1274	1.84E-04	2.96E-02	1.6934
**1628155_at**	38067	klar	1.0622	9.3789	11.1234	1.84E-04	2.96E-02	1.6918
**1633795_a_at**	34485	CG17124	-1.1641	9.0199	-11.0953	1.86E-04	2.96E-02	1.6808
**1633059_at**	36532	CG6357	2.6408	9.1864	11.0697	1.88E-04	2.97E-02	1.6707
**1636091_at**	37089	fj	2.4363	6.1064	10.9906	1.94E-04	2.97E-02	1.6393
**1632790_at**	35528	dream	1.4360	6.1042	10.9592	1.97E-04	2.97E-02	1.6267
**1640904_at**	39394	thoc6	1.0437	5.2542	10.9520	1.97E-04	2.97E-02	1.6239
**1623753_at**	34045	TepIII	2.0456	4.7897	10.8916	2.02E-04	3.01E-02	1.5996
**1639292_at**	32797	Frq1	-1.9796	5.5936	-10.7218	2.16E-04	3.05E-02	1.5304
**1638556_s_at**	43982	Oamb	-1.1816	4.7866	-10.7160	2.17E-04	3.05E-02	1.5280
**1640440_at**	44324	Dms	-1.5278	9.2515	-10.7057	2.18E-04	3.05E-02	1.5238
**1631246_at**	36030	Fmrf	-1.9798	6.7802	-10.6374	2.24E-04	3.05E-02	1.4955
**1633530_at**	53446	HGTX	-1.0637	4.0343	-10.6280	2.25E-04	3.05E-02	1.4916
**1636057_at**	33013	CG9572	1.8197	7.1457	10.6128	2.26E-04	3.05E-02	1.4853
**1630477_at**	38779	msl-3	1.5901	6.4588	10.5893	2.28E-04	3.05E-02	1.4755
**1628632_at**	41591	Paip2	-2.5355	7.6578	-10.5329	2.34E-04	3.08E-02	1.4518
**1625012_s_at**	34652	vir-1	1.2422	10.0148	10.5217	2.35E-04	3.08E-02	1.4471
**1630986_s_at**	41318	Adk3	-1.0335	7.3240	-10.4419	2.43E-04	3.13E-02	1.4134
**1639850_at**	31043	CG3704	1.0849	8.2710	10.4419	2.43E-04	3.13E-02	1.4133
**1625616_at**	40421	CG14566	-2.4900	5.5315	-10.4274	2.44E-04	3.13E-02	1.4072
**1632873_at**	41202	MtnA	-1.7564	11.8708	-10.3681	2.51E-04	3.15E-02	1.3818
**1622901_at**	37358	CG9993	1.2411	6.0047	10.2894	2.59E-04	3.19E-02	1.3479
**1629269_at**	317913	CG32204	-1.1435	6.5596	-10.2416	2.64E-04	3.19E-02	1.3272
**1633696_at**	37786	TM4SF	-1.1223	8.5756	-10.2021	2.69E-04	3.19E-02	1.3099
**1640978_at**	40420	CG14567	2.2234	6.5034	10.1617	2.74E-04	3.20E-02	1.2922
**1641230_at**	34538	Ast-CC	-1.3381	4.4352	-10.1328	2.77E-04	3.20E-02	1.2795
**1625114_at**	35115	Cyp310a1	1.1847	5.1208	9.9949	2.94E-04	3.29E-02	1.2182
**1634707_s_at**	3354921	Gfat1	1.8623	7.7075	9.9760	2.97E-04	3.29E-02	1.2097
**1639594_at**	3355165	CG40485	2.9464	4.6908	9.9570	2.99E-04	3.29E-02	1.2011
**1632543_at**	36244	CG9003	-1.1730	7.6540	-9.9361	3.02E-04	3.29E-02	1.1917
**1638581_at**	32977	Ubqn	1.1622	8.5310	9.9349	3.02E-04	3.29E-02	1.1911
**1639183_a_at**	42379	mira	2.5401	10.1449	9.9220	3.04E-04	3.29E-02	1.1853
**1625512_s_at**	3885644	CG34002	1.2494	4.5307	9.9159	3.05E-04	3.29E-02	1.1825
**1632177_at**	41273	hth	-2.6040	5.7193	-9.8477	3.14E-04	3.36E-02	1.1516
**1641476_a_at**	41248	Timp	1.9976	6.3388	9.6509	3.43E-04	3.55E-02	1.0606
**1640944_at**	33291	CG4577	-1.0974	7.0582	-9.6370	3.45E-04	3.55E-02	1.0541

**1625719_at**	48971	Atpalpha	-1.2520	9.2240	-9.5813	3.54E-04	3.60E-02	1.0279
**1636927_at**	246578	CG30379	-1.8190	5.3031	-9.5131	3.65E-04	3.63E-02	0.9957
**1628146_at**	42896	crb	1.2068	6.8303	9.4818	3.70E-04	3.63E-02	0.9807
**1637412_a_at**	40861	sas	2.1444	6.0684	9.4711	3.72E-04	3.63E-02	0.9756
**1638616_at**	38056	mthl9	2.3906	5.0559	9.4708	3.72E-04	3.63E-02	0.9755
**1626804_at**	42625	CG5379	-1.1717	3.9267	-9.4429	3.77E-04	3.66E-02	0.9621
**1638132_at**	42783	CG10184	-1.7837	5.4193	-9.3769	3.88E-04	3.72E-02	0.9303
**1632980_at**	40059	CG3902	-1.2833	8.1154	-9.3116	4.00E-04	3.79E-02	0.8986
**1637705_at**	30975	ewg	1.0479	7.2496	9.2919	4.04E-04	3.79E-02	0.8890
**1623810_at**	33509	CG17265	1.5809	6.4141	9.1377	4.35E-04	3.92E-02	0.8129
**1626606_at**	38620	CG10630	-1.3937	3.6764	-9.1359	4.35E-04	3.92E-02	0.8120
**1638870_at**	31661	CG1958	-1.1225	4.4495	-9.0821	4.46E-04	3.95E-02	0.7851
**1630130_at**	32241	CG4404	1.8263	5.5871	9.0452	4.54E-04	3.97E-02	0.7665
**1629551_s_at**	42833	CG12268	1.1942	4.4710	9.0407	4.55E-04	3.97E-02	0.7642
**1636059_at**	31922	CG9689	2.8070	5.7024	8.9868	4.67E-04	4.06E-02	0.7370
**1640227_at**	35963	CG8801	1.1629	10.7630	8.9187	4.83E-04	4.15E-02	0.7022
**1634350_at**	33581	sob	1.7774	4.7546	8.8543	4.98E-04	4.15E-02	0.6691
**1638097_at**	3346207	CG33543	-1.2781	5.4619	-8.8241	5.05E-04	4.15E-02	0.6534
**1641428_at**	37941	Cyp9c1	-1.5850	5.4134	-8.8235	5.06E-04	4.15E-02	0.6531
**1639928_a_at**	36740	Zasp52	2.2423	6.1897	8.8165	5.07E-04	4.15E-02	0.6495
**1631931_s_at**	37447	Sdc	-1.2980	8.3560	-8.7733	5.18E-04	4.15E-02	0.6270
**1627167_a_at**	3355165	CG40485	2.3945	5.7176	8.7726	5.18E-04	4.15E-02	0.6267
**1624745_at**	2768992	Ilp5	-3.1592	7.4847	-8.7624	5.21E-04	4.15E-02	0.6213
**1628252_at**	40780	CG17919	1.1669	6.5092	8.7481	5.25E-04	4.15E-02	0.6139
**1636835_at**	32694	CG16700	-1.1906	6.2855	-8.7443	5.26E-04	4.15E-02	0.6119
**1628585_at**	42001	Gyc-89Da	-1.3019	3.8715	-8.7437	5.26E-04	4.15E-02	0.6115
**1640979_at**	32299	CG1681	1.0547	7.6620	8.7186	5.32E-04	4.17E-02	0.5983
**1639883_at**	45307	fz	-1.0876	5.1637	-8.6958	5.38E-04	4.18E-02	0.5864
**1629745_at**	42586	CG6439	-1.3997	8.1527	-8.6925	5.39E-04	4.18E-02	0.5846
**1623016_at**	38496	CG1299	1.2698	6.3028	8.6864	5.41E-04	4.18E-02	0.5814
**1641118_at**	43936	Mdh	-1.0293	7.8590	-8.6239	5.58E-04	4.25E-02	0.5483
**1633582_at**	36589	Ih	-1.7309	7.2304	-8.6236	5.58E-04	4.25E-02	0.5481
**1641370_s_at**	31174	CG4199	1.3005	8.3305	8.6096	5.62E-04	4.26E-02	0.5406
**1623315_at**	40288	CG13253	-1.5802	6.1690	-8.5914	5.67E-04	4.28E-02	0.5310
**1632533_at**	41248	Timp	1.3152	6.7677	8.5105	5.91E-04	4.36E-02	0.4875
**1631378_at**	34947	beat-Ia	-1.2760	6.3497	-8.5052	5.92E-04	4.36E-02	0.4847
**1635175_at**	42721	CG17121	1.8622	6.3135	8.4097	6.22E-04	4.52E-02	0.4328
**1639042_at**	31352	CG6414	2.5349	5.1646	8.3792	6.32E-04	4.55E-02	0.4160
**1640377_s_at**	31332	Rala	1.7431	7.8144	8.3462	6.43E-04	4.57E-02	0.3979
**1630653_a_at**	32087	Gs2	-2.0144	9.3476	-8.3021	6.57E-04	4.57E-02	0.3735
**1637378_s_at**	33277	ia2	-1.7094	8.6920	-8.2987	6.58E-04	4.57E-02	0.3716
**1636046_at**	33144	Cda4	2.1228	6.4746	8.2947	6.60E-04	4.57E-02	0.3694
**1641324_at**	38723	LanA	1.0723	8.6798	8.2833	6.64E-04	4.57E-02	0.3630

Biology Open • Supplementary information

**1625154_s_at**	32154	pot	2.8899	7.1894	8.2739	6.67E-04	4.57E-02	0.3578
**1633280_s_at**	40522	CG12581	1.8080	7.0664	8.2735	6.67E-04	4.57E-02	0.3575
**1637823_at**	32377	CG1434	1.1927	6.6448	8.1831	6.99E-04	4.63E-02	0.3069
**1633427_at**	43492	CG7582	-1.0546	6.2754	-8.1778	7.01E-04	4.63E-02	0.3039
**1635500_a_at**	41363	pros	2.6178	9.6835	8.1703	7.04E-04	4.63E-02	0.2997
**1624488_a_at**	41359	CG17734	-1.6036	9.6428	-8.0981	7.31E-04	4.74E-02	0.2588
**1626513_at**	40083	MESR6	-1.0744	8.1781	-8.0955	7.32E-04	4.74E-02	0.2573

Table S3: Genes significantly associated with GO-terms

GO-ID	corr p-value	Description	Genes in test set
48513	1.47E-04	organ development	*Ddc|ewg|by|Mdh|mir-1|mir-317|klar|odd|grh|ft|Tsp|mir-9a|hth|LanA|cas|Rala|CG13253|pros*
9887	2.12E-04	organ morphogenesis	*ewg|by|mir-317|klar|grh|odd|ft|hth|mir-9a|cas|LanA|Rala|pros*
9653	2.07E-03	anatomical structure morphogenesis	*ewg|by|let-7|Mdh|mir-317|klar|odd|grh|ft|mir-9a|hth|scrt|LanA|cas|Rala|exba|pros*
32502	2.07E-03	developmental process	*Ddc|Zasp52|ewg|by|let-7|stv|Mdh|mir-1|mir-317|klar|odd|grh|ft|Tsp|sas|mir-9a|hth|scrt|LanA|cas|Rala|CG13253|exba|pros*
7402	2.08E-03	ganglion mother cell fate determination	*cas|pros|grh*
48731	2.45E-03	system development	*Ddc|ewg|by|Mdh|mir-1|mir-317|klar|odd|grh|ft|Tsp|mir-9a|hth|scrt|LanA|cas|Rala|CG13253|exba|pros*
48856	3.05E-03	anatomical structure development	*Zasp52|Ddc|ewg|by|let-7|Mdh|mir-1|mir-317|klar|odd|grh|ft|Tsp|mir-9a|hth|scrt|LanA|cas|Rala|CG13253|exba|pros*
48663	3.05E-03	neuron fate commitment	*hth|Rala|exba|pros|grh*
50793	3.51E-03	regulation of developmental process	*Ddc|ewg|ft|let-7|hth|LanA|cas|Rala|pros|grh*
32501	4.49E-03	multicellular organismal process	*Ddc|ewg|by|Oamb|Mdh|mir-1|mir-317|klar|vis|odd|grh|ft|Tsp|sas|ir76a|mir-9a|hth|scrt|LanA|cas|Rala|CG13253|exba|pros*
7275	5.34E-03	multicellular organismal development	*Ddc|ewg|by|Mdh|mir-1|mir-317|klar|odd|grh|ft|Tsp|sas|mir-9a|hth|scrt|LanA|cas|Rala|CG13253|exba|pros*
7417	5.65E-03	central nervous system development	*ewg|hth|LanA|cas|mir-317|pros|grh*
7552	9.68E-03	metamorphosis	*by|ewg|ft|let-7|mir-9a|hth|cas|Mdh|odd*
65007	1.50E-02	biological regulation	*Ddc|ewg|by|let-7|Oamb|Mdh|mir-1|klar|vis|odd|grh|ft|ir76a|mir-9a|hth|scrt|msl-3|LanA|cas|Rala|CG13253|exba|pros*
7420	1.50E-02	brain development	*hth|LanA|cas|mir-317|pros*
5924	1.50E-02	cell-substrate adherens junction	*Zasp52|by|Tsp*
30055	1.50E-02	cell-substrate junction	*Zasp52|by|Tsp*
1752	1.50E-02	compound eye photoreceptor fate commitment	*hth|Rala|pros|grh*
42706	1.50E-02	eye photoreceptor cell fate commitment	*hth|Rala|pros|grh*
2165	1.50E-02	instar larval or pupal development	*by|ewg|ft|sas|mir-9a|hth|cas|Mdh|odd*
1071	1.50E-02	nucleic acid binding transcription factor activity	*ewg|scrt|hth|cas|pros|vis|odd|grh*
46552	1.50E-02	photoreceptor cell fate commitment	*hth|Rala|pros|grh*
3700	1.50E-02	sequence-specific DNA binding transcription factor activity	*ewg|scrt|hth|cas|pros|vis|odd|grh*
9791	1.64E-02	post-embryonic development	*by|ewg|ft|sas|mir-9a|hth|cas|Mdh|odd*
1751	1.72E-02	compound eye photoreceptor cell	*hth|Rala|klar|pros|grh*

Biology Open • Supplementary information

		differentiation	
5927	1.72E-02	muscle tendon junction	*Zasp52|Tsp*
1754	1.82E-02	eye photoreceptor cell differentiation	*hth|Rala|klar|pros|grh*
48707	1.82E-02	instar larval or pupal morphogenesis	*by|ewg|ft|mir-9a|hth|cas|Mdh|odd*
9886	1.99E-02	post-embryonic morphogenesis	*by|ewg|ft|mir-9a|hth|cas|Mdh|odd*
45165	2.17E-02	cell fate commitment	*stv|hth|cas|Mdh|Rala|exba|pros|grh*
1745	2.42E-02	compound eye morphogenesis	*ft|hth|Rala|klar|pros|grh*
7560	2.42E-02	imaginal disc morphogenesis	*by|ewg|ft|mir-9a|hth|cas|odd*
48563	2.42E-02	post-embryonic organ morphogenesis	*by|ewg|ft|mir-9a|hth|cas|odd*
7419	2.42E-02	ventral cord development	*cas|pros|grh*
46530	2.45E-02	photoreceptor cell differentiation	*hth|Rala|klar|pros|grh*
48592	3.03E-02	eye morphogenesis	*ft|hth|Rala|klar|pros|grh*
5886	3.05E-02	plasma membrane	*Zasp52|by|ft|Tsp|sas|Oamb|Rala|tm4sf|pros*
48569	3.05E-02	post-embryonic organ development	*by|ewg|ft|mir-9a|hth|cas|odd*
50789	3.13E-02	regulation of biological process	*Ddc|ewg|let-7|Oamb|Mdh|mir-1|vis|odd|grh|ft|ir76a|mir-9a|hth|scrt|msl-3|LanA|cas|Rala|CG13253|exba|pros*
16323	3.64E-02	basolateral plasma membrane	*Zasp52|by|Tsp*
48699	3.64E-02	generation of neurons	*scrt|hth|LanA|cas|Rala|klar|exba|pros|grh*
7444	3.64E-02	imaginal disc development	*Ddc|by|ewg|ft|mir-9a|hth|cas|odd*
35120	3.64E-02	post-embryonic appendage morphogenesis	*by|ft|mir-9a|hth|cas|odd*
6357	3.64E-02	regulation of transcription from RNA polymerase II promoter	*scrt|hth|cas|pros|vis|odd|grh*
35107	3.83E-02	appendage morphogenesis	*by|ft|mir-9a|hth|cas|odd*
48859	3.83E-02	formation of anatomical boundary	*ft|mir-9a|hth*
35114	3.83E-02	imaginal disc-derived appendage morphogenesis	*by|ft|mir-9a|hth|cas|odd*
48519	3.83E-02	negative regulation of biological process	*ewg|ft|mir-9a|scrt|cas|Rala|CG13253|exba|pros|odd*
40034	3.83E-02	regulation of development, heterochronic	*let-7|cas*
48737	3.87E-02	imaginal disc-derived appendage development	*by|ft|mir-9a|hth|cas|odd*
48736	3.92E-02	appendage development	*by|ft|mir-9a|hth|cas|odd*
10468	4.50E-02	regulation of gene expression	*ewg|mir-9a|scrt|hth|msl-3|cas|exba|pros|vis|odd|grh*
71944	4.66E-02	cell periphery	*Zasp52|by|ft|Tsp|sas|Oamb|Rala|tm4sf|pros*
48749	4.66E-02	compound eye development	*ft|hth|Rala|klar|pros|grh*
30182	4.66E-02	neuron differentiation	*scrt|hth|LanA|Rala|klar|exba|pros|grh*
5515	4.66E-02	protein binding	*Zasp52|by|stv|klar|vis|grh|ft|hth|LanA|msl-3|Rala|CG13253|exba*
1964	4.76E-02	startle response	*LanA|mir-317*

Biology Open • Supplementary information

48523	4.78E-02	negative regulation of cellular process	*ewg|ft|scrt|cas|Rala|CG13253|exba|pros|odd*
7423	4.94E-02	sensory organ development	*ft|mir-9a|hth|Rala|klar|pros|grh*
